# A surge of cytosolic calcium dysregulates lysosomal function and impairs autophagy flux during cupric chloride–induced neuronal death

**DOI:** 10.1016/j.jbc.2023.105479

**Published:** 2023-11-21

**Authors:** Yoonkyung Kim, Yangsin Lee, Minjung Choo, Nuri Yun, Jin Won Cho, Young J. Oh

**Affiliations:** 1 Department of Systems Biology Yonsei University College of Life Science and Biotechnology, Seoul, Korea; 2 Glycosylation Network Research Center, Yonsei University, Seoul, Korea; 3 GNT Pharma Science Technology Center for Health, Incheon, Korea

**Keywords:** autophagy, copper, cytosolic calcium, lysosome, neurodegeneration

## Abstract

Autophagy is a degradative pathway that plays an important role in maintaining cellular homeostasis. Dysfunction of autophagy is associated with the progression of neurodegenerative diseases including Alzheimer’s disease, Parkinson’s disease, and amyotrophic lateral sclerosis. Although one of the typical features of brain aging is an accumulation of redox-active metals that eventually lead to neurodegeneration, a plausible link between trace metal-induced neurodegeneration and dysregulated autophagy has not been clearly determined. Here, we used a cupric chloride-induced neurodegeneration model in MN9D dopaminergic neuronal cells along with ultrastructural and biochemical analyses to demonstrate impaired autophagic flux with accompanying lysosomal dysfunction. We found that a surge of cytosolic calcium was involved in cupric chloride-induced dysregulated autophagy. Consequently, buffering of cytosolic calcium by calbindin-D28K overexpression or co-treatment with the calcium chelator BAPTA attenuated the cupric chloride-induced impairment in autophagic flux by ameliorating dysregulation of lysosomal function. Thus, these events allowed the rescue of cells from cupric chloride-induced neuronal death. These phenomena were largely confirmed in cupric chloride-treated primary cultures of cortical neurons. Taken together, these results suggest that abnormal accumulation of trace metal elements and a resultant surge of cytosolic calcium leads to neuronal death by impairing autophagic flux at the lysosomal level.

Autophagy is a process that removes unnecessary or dysfunctional cellular components and plays a major role in cell homeostasis (1). Among three major types of autophagy, macroautophagy (hereafter referred to as autophagy) is activated in response to cellular stresses. When cells encounter stressful situations, autophagy initiates with the formation of the autophagosome that subsequently sequesters a portion of the cytoplasm including damaged organelles. To degrade its cargo, autophagosomes fuse with lysosomal membranes to form autolysosomes (2). Therefore, autophagy is an important catabolic process for maintaining homeostatic metabolism. Consequently, excessive or insufficient autophagy is associated with the pathogenesis of various diseases accompanying cell death (3, 4). Accumulating evidence indicates that dysregulated autophagy is involved in neurodegenerative diseases including Alzheimer’s disease (AD), Parkinson’s disease (PD), amyotrophic lateral sclerosis (ALS), and Huntington’s disease (HD) (5, 6, 7, 8, 9). Furthermore, the interplay between autophagy and other cell death processes was recently discovered to be connected to the progression of neurodegeneration (4, 10, 11, 12).

The etiology of neurodegenerative diseases is incompletely understood, although several different possibilities have been proposed (5, 13). Recent studies indicate that mutations in disease-related genes cause familial forms of diseases, but most cases are sporadic (14). An increasing body of evidence indicates that oxidative stress, mitochondrial dysfunction, inflammation, impairment of the ubiquitin-proteasome system, and autophagy comprise a unifying mechanism underlying the pathophysiology of both familiar and sporadic cases. Moreover, the environment is also a source of putative risk factors (15). For example, pre- and postnatal exposure to environmental factors, including neurotoxic metals, pesticides, and metal-based nanoparticles, increases the risk of developing neurodegenerative diseases later in life. Most heavy metals are found in nature and are essential compounds in the human body. However, even low levels of exposure to heavy metals can lead to cytotoxicity (16). Based on their ability to increase abnormal protein aggregates, altered homeostasis of heavy metals is proposed to contribute to AD and PD pathogenesis (17). ALS patients also have higher metal levels than age-matched controls (18). Regarding potential underlying mechanisms, reactive oxygen species (ROS)- and Fas/FasL-mediated apoptosis are observed in metal-exposed neuronal cells (19, 20). More recently, methylmercury was found to induce autophagy *via* the JNK/Vps34 complex pathway and promote autophagosome accumulation and neuronal cell death (21). Therefore, the balance between acquisition and distribution is tightly regulated to protect against heavy metal-mediated cytotoxicity.

Copper (Cu) is an inorganic element that is essential for various species (22). For example, Cu plays an important role in cellular processes including gene transcription, energy metabolism, neurotransmitter biosynthesis, and antioxidant defense (23). In the nervous system, Cu is involved in myelination, synaptic activity, signaling cascades, and excitotoxicity (24). Cu can pass through the endothelial membrane of the blood-brain barrier (BBB) *via* transporters such as CTR1, ATP7A, and ATP7B (23). However, excess levels of Cu are observed in several neurodegenerative diseases (25). Excess Cu levels in patients with AD are proposed to cause damage to the BBB and bind to amyloid β with high affinity, eventually leading to accumulation of amyloid β plaque, microglial and astroglial activation, and neuroinflammation (23, 26, 27, 28). In PD, excess Cu leads to α-synuclein aggregation and eventually neuronal cell death by increasing oxidative stress and inducing mitochondrial dysfunction (24, 29). Overexpression of α-synuclein at non-toxic levels can cause neuronal cell death in the presence of Cu ions (30). Similar patterns of Cu-induced neurotoxicity are observed in other neurodegenerative diseases including ALS and stroke (24, 27). Consequently, several therapeutic strategies are proposed to reverse homeostatic disturbances in brain Cu levels (24).

Recent progress in studying Cu-mediated signaling has been made by exploiting Cu-dependent disease vulnerabilities (31, 32). A diverse array of mediating cellular processes has been proposed. Of these, Cu-induced autophagy is observed in various cell types including hepatocytes (33), monocytes (34), male germ cells (35, 36), and renal tubular epithelial cells (37). Despite this recent progress in understanding Cu-mediated cytotoxicity, whether and how autophagic flux is associated with Cu-induced neurotoxicity has not been thoroughly determined. In the present study, therefore, we specifically asked whether and how a cupric chloride (CuCl_2_)-induced surge of ROS and cytosolic Ca^2+^ causes dysregulated autophagy. Here, we found that a CuCl_2_-induced surge of cytosolic Ca^2+^ but not ROS is responsible for dysregulated autophagic flux and resultant neuronal death. As this event is caused by lysosomal deficits, the buffering of cytosolic Ca^2+^ *via* overexpression of a Ca^2+^-binding protein (calbindin D28K) or calcium chelator (BAPTA-AM) protects cells against CuCl_2_-induced death by preserving the autophagy-lysosome pathway.

## Results

### Characteristic features of CuCl_2_-induced autophagy in MN9D cells

**Figure 1:**
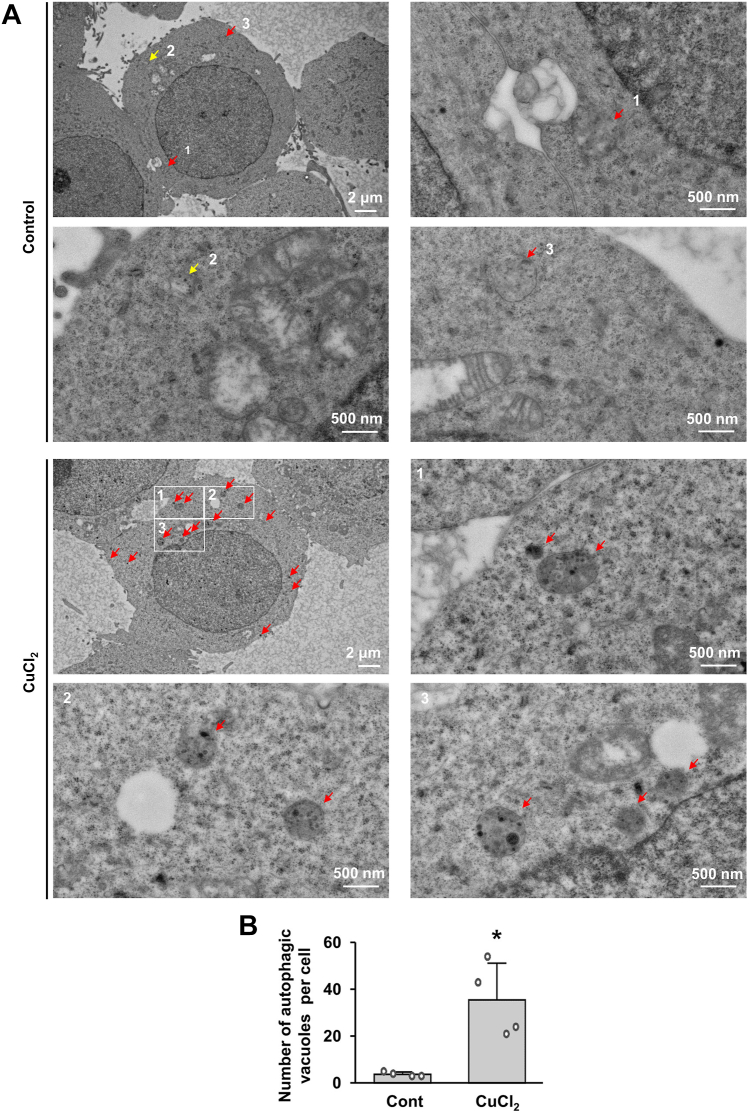
**Ultrastructural characterization of autophagy in MN9D cells following CuCl**_**2**_**treatment.***A*, transmission electron micrographs were taken after treatment with or without 250 μM CuCl_2_ for 15 h. Scale bar represents 2 μm or 500 nm. Enlarged images of numbered areas illustrate typical autophagic vacuoles (*red arrows*) and multivesicular bodies (*yellow arrows*). *B*, the number of autophagic vacuoles per cell was quantified in four randomly selected cells per group. Data are shown as the mean ± S.D. ∗*p* < 0.05.

To characterize CuCl_2_-induced cell death in MN9D cells, we examined changes in morphology and rate of cell death using phase-contrast microscopy and 3-(4,5-dimethylthiazol-2-yl)-2,5-diphenyltetrazolium bromide (MTT) reduction assay, respectively. Compared with non-treated control cells, CuCl_2_-treated cells showed morphological features of shrinkage of cell bodies and retraction of neurites (Fig. S1*A*). The MTT reduction assay indicated that CuCl_2_ induced dramatic cell death by 15 h (Fig. S1*B*). Furthermore, ultrastructural changes observed by transmission electron microscopy showed that CuCl_2_ treatment increased the appearance of autophagic vacuoles, including double-membrane autophagosomes and single-membrane autolysosomes (Fig. 1*A*). To quantify autophagic vacuoles, we applied stringent criteria including the presence of partial or complete double membrane-bound vacuoles, contents within membrane-bound vacuoles, and electron density inside vacuoles. Using these criteria, we scrutinized images of serial sections of cells to identify autophagic vacuoles as typified in Fig. S2. We found that the number of autophagic vacuoles was markedly increased following CuCl_2_ treatment (Fig. 1*B*).

**Figure 2:**
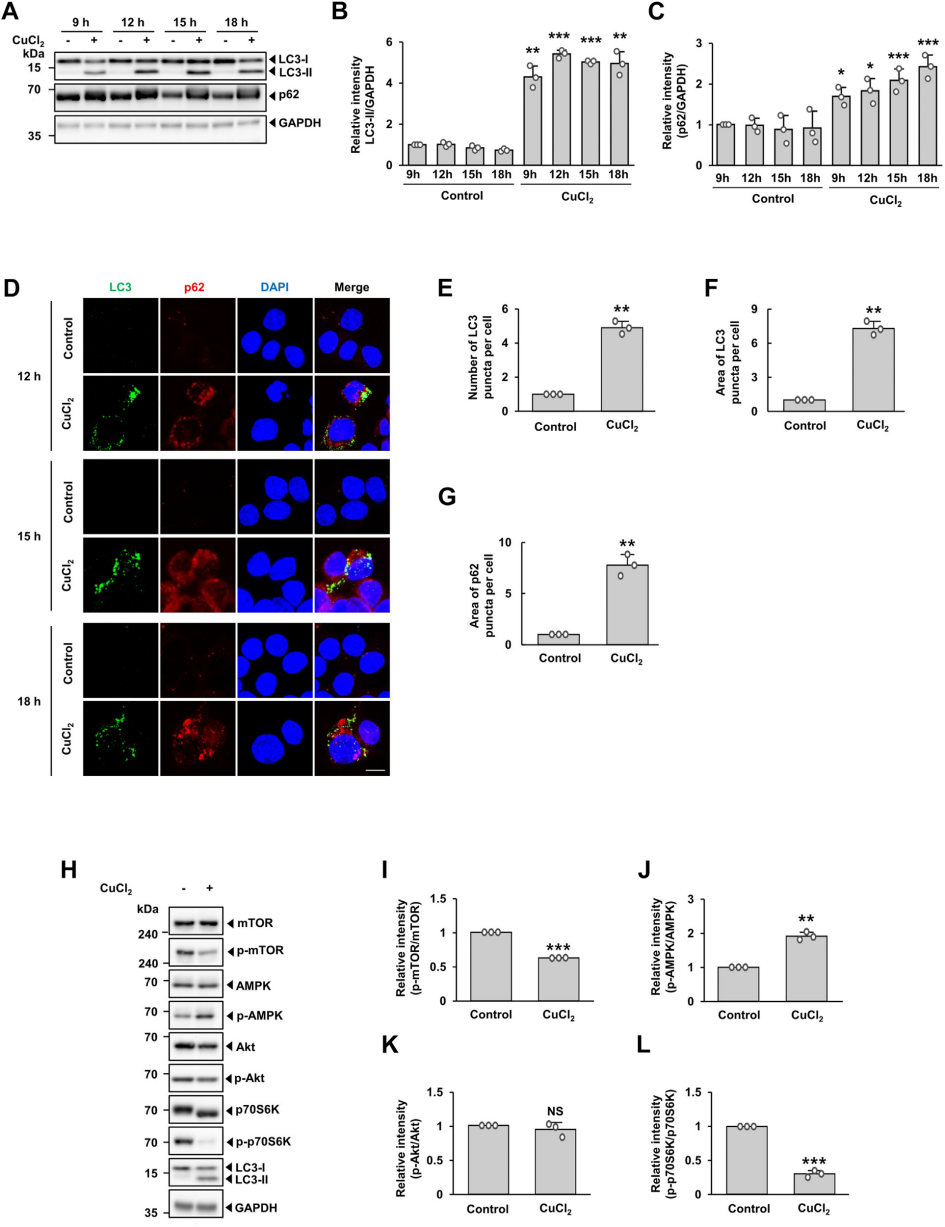
**Biochemical features of autophagy in MN9D cells treated with CuCl**_**2**_**.** Cells were treated with or without 250 μM CuCl_2_ for the indicated time periods. *A*, immunoblot analyses were performed using anti-LC3 or anti-p62 antibodies. Anti-GAPDH antibody was utilized as a loading control. The relative intensities of LC3-II (*B*) and p62 (*C*) signals at the indicated time points were measured using ImageJ software, normalized by GAPDH signal, and expressed as fold change relative to untreated control (value = 1). Data are shown as the mean ± S.D of three independent experiments. ∗*p* < 0.05; ∗∗*p* < 0.01; ∗∗∗*p* < 0.001. *D*, cells treated with 250 μM CuCl_2_ for 15 h were subjected to immunocytochemical localization of LC3 (*green*) and p62 (*red*). Nuclei were counterstained with Hoechst 33258 (*blue*). Cells were examined under a confocal microscope. Merged images are shown to the *right*. Scale bar represents 10 μm. The number (*E*) and area (*F*) of LC3 and the area of p62 (*G*) puncta per cell were quantified using ImageJ software. Data are expressed as fold change relative to untreated control (value = 1) and shown as the mean ± S.D of three independent experiments. ∗∗*p* < 0.01. *H*, cellular lysates were subjected to immunoblot analyses using the indicated antibodies. After normalization against the intensity of total protein, the relative intensities of the phosphorylated forms of mTOR (*I*), p-AMPK (*J*), p-Akt (*K*), and p-p70S6K (*L*) are expressed as fold change relative to untreated controls (value = 1). Data are shown as the mean ± S.D of three independent experiments. ∗∗*p* < 0.01; ∗∗∗*p* < 0.001; NS, not significant.

Among several molecular markers of autophagy studied to date, conversion of the cytosolic form of LC3-1 to LC3-II *via* phosphatidylethanolamine conjugation is the standard indicator of autophagosome formation. p62/SQSTM1 is also widely used as an autophagic marker as it is an autophagosome cargo protein that binds to target proteins (38). Immunoblot analyses indicated that, following CuCl_2_ treatment, generation of LC3-II appeared as early as 9 h and plateaued thereafter (Fig. 2, *A* and *B*). This increase in LC3-II paralleled the increased expression of p62 (Fig. 2, *A* and *C*). Immunofluorescence analyses showed that the staining pattern of LC3 and p62 changed from diffused to punctate in MN9D cells treated with CuCl_2_ (Fig. 2*D*). At 15 h, quantitative analyses indicated that LC3 puncta number and area, and p62 puncta area were significantly increased in CuCl_2_-treated cells compared with non-treated control cells (Fig. 2, *E*–*G*). To obtain additional evidence of autophagy activation following CuCl_2_ treatment, we performed immunoblot analyses to determine whether autophagy induced by CuCl_2_ affects the AKT/mTOR and/or AMPK pathway (39, 40) (Fig. 2*H*). Quantitative analyses of immunoblots indicated that phosphorylated forms of mTOR and an mTOR substrate, p70S6K, decreased after CuCl_2_ treatment (Fig. 2, *I* and *L*), whereas AMPK activity increased (Fig. 2*J*). Intriguingly, Akt activity was not altered by CuCl_2_ treatment (Fig. 2*K*). In sum, our biochemical and morphological data indicate that autophagy is activated in MN9D cells following CuCl_2_ treatment.

### Dysregulated autophagy induced by CuCl_2_ treatment

**Figure 3:**
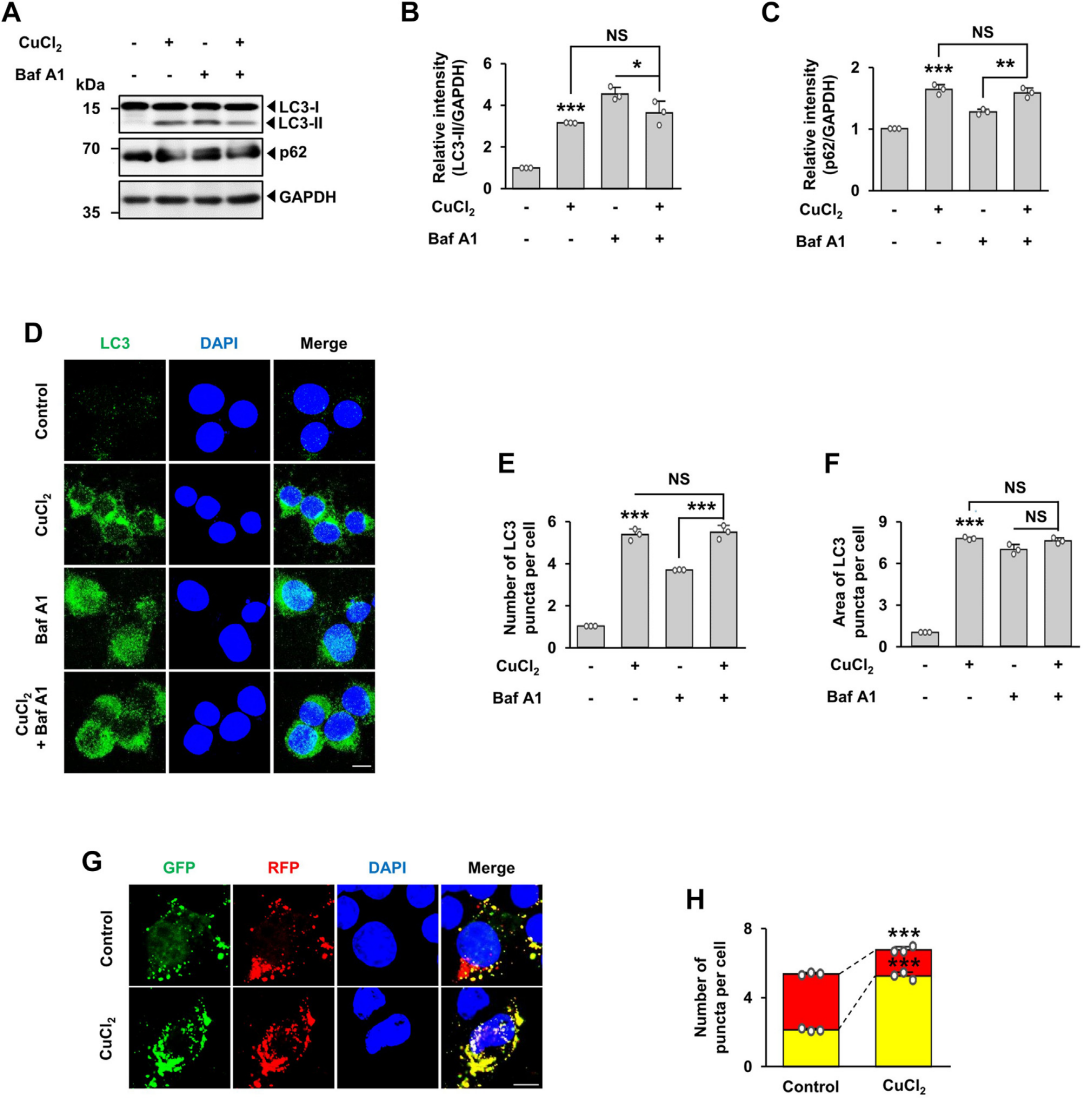
**CuCl**_**2**_**-mediated blockade of autophagy flux.** MN9D cells were treated with or without 250 μM CuCl_2_ for 15 h in the presence or absence of 25 nM Baf.A1 for the final 6 h. *A*, cell lysates were subjected to autophagy flux assay by monitoring and comparing levels of LC3-II and p62. The relative intensity of normalized LC3-II (*B*) and p62 (*C*) signals measured using ImageJ software expressed as fold change relative to untreated control (value = 1). Data are shown as the mean ± S.D of three independent experiments. Two-way ANOVA followed by Tukey’s post hoc test was performed. ∗*p* < 0.05; ∗∗*p* < 0.01; ∗∗∗*p* < 0.001; NS, not significant. *D*, immunocytochemical analyses were performed using anti-LC3 (*green*) followed by nuclei counterstaining with Hoechst 33258 (*blue*). Fluorescent images were obtained from the confocal examination. Merged images are shown to the *right*. Scale bar represents 10 μm. The number (*E*) and area (*F*) of LC3 puncta per cell were quantified using ImageJ software. Data are expressed as fold change relative to untreated control (value = 1) and shown as the mean ± S.D of three independent experiments. Two-way ANOVA followed by Tukey’s post hoc test was performed. ∗∗∗*p* < 0.001; NS, not significant. *G*, MN9D cells were transfected with an mRFP-EGFP–tagged LC3B probe for 24 h and then treated with 250 μM CuCl_2_ for 15 h. After fixation, nuclei were counterstained with Hoechst 33258 (*blue*). Fluorescent images were acquired using confocal microscopy. Merged images are shown to the *right*. Scale bar represents 10 μm. *H*, quantification of the number of *yellow* (mRFP^+^-EGFP^+^) and *red* (mRFP^+^-EGFP^−^) puncta from the merged images was performed using ImageJ software. Data are shown as the mean ± S.D of three independent experiments. ∗∗∗*p* < 0.001.

Autophagic flux is a measure of autophagic activity starting from the formation of autophagosomes to the degradation of autophagic substrates. Accumulation of autophagosomes and accompanying increased levels of LC3-II and p62 can be indicative of either activation of autophagic induction or impaired autophagic flux (1, 41). To determine whether CuCl_2_-induced changes of LC3 and p62 are a consequence of activation of autophagy or the blockade of autophagic flux at lysosome-mediated degradation events, we monitored autophagy flux following co-treatment with Bafilomycin A1 (Baf.A1), an inhibitor of lysosomal acidification and autolysosomal activity. Among several of widely used lysosomal inhibitors (*e.g.*, chloroquine), usage of Baf.A1 was empirically chosen. Immunoblot analyses indicated that co-treatment with CuCl_2_ and Baf.A1 did not further enhance levels of LC3-II and p62 compared with levels in cells treated only with CuCl_2_ (Fig. 3, *A*–*C*). Consistent with these findings, immunofluorescence assay indicated that the number and area of LC3 puncta were similar between cells treated with CuCl_2_ with or without Baf.A1 (Fig. 3, *D*–*F*), suggesting that CuCl_2_-induced accumulation of LC3-II and p62 was due to a blockade of autophagic flux at the lysosomal level. To further test our hypothesis, we used a tandem fluorescent-tagged LC3 (mRFP-EGFP-tagged LC3). This probe is widely used for monitoring autophagic flux based on different pH stability of mRFP and EGFP fluorescent proteins (38, 41). EGFP fluorescence is quenched in acidic environments or acidic lysosomes whereas mRFP is more stable in low pH environment. Therefore, a tandem mRFP-EGFP-tagged LC3 located in autophagosome show yellow puncta because both mRFP and EGFP fluorescence is observed. By contrast, a tandem mRFP-EGFP-tagged LC3 located in autolysosome show red puncta because EGFP fluorescence is quenched and only mRFP retain its fluorescence. CuCl_2_ treatment increased the number of yellow puncta (EGFP^+^/mRFP^+^) and decreased the number of red puncta (EGFP^−^/mRFP^+^) compared with the control group (Fig. 3, *G* and *H*), confirming that CuCl_2_ treatment impairs autophagic flux at the lysosomal level.

### Lysosomal dysfunction induced by CuCl_2_ treatment

**Figure 4:**
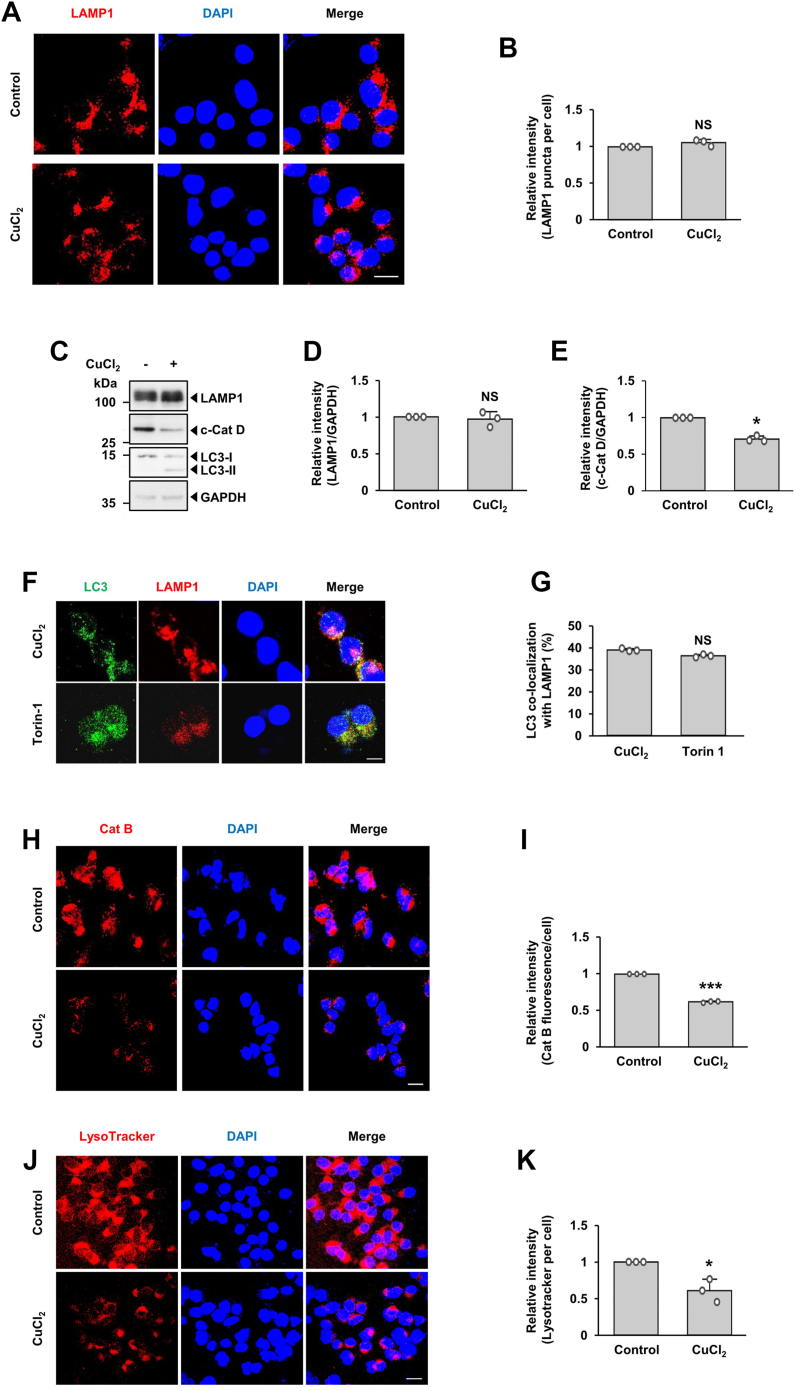
**Lysosomal dysfunction induced by CuCl**_**2**_**treatment.** MN9D cells were treated with or without 250 μM CuCl_2_ for 15 h. *A*, immunocytochemical analyses were performed using anti-LAMP1 (*red*) followed by nuclei counterstaining with Hoechst 33258 (*blue*). Merged images are shown to the *right*. Scale bar represents 20 μm. *B*, the number of LAMP1 puncta per cell was quantified using ImageJ software. Data are shown as the mean ± S.D of three independent experiments. NS, not significant. *C*, immunoblot analyses were performed using anti-LAMP1, anti-cathepsin D, or anti-LC3 antibodies. The cleaved band of cathepsin D (c-Cat D) was detected by an anti-cathepsin D antibody. The relative intensities of LAMP1 (*D*) and c-Cat D (*E*) signals were measured using ImageJ software, normalized by GAPDH signal, and expressed as fold change relative to untreated control (value = 1). Data are shown as the mean ± S.D of three independent experiments. ∗*p* < 0.05; NS, not significant. *F*, after treatment with 250 μM CuCl_2_ for 15 h or 500 nM Torin-1 for 24 h, cells were subjected to immunocytochemical analyses after probing with anti-LC3 (*green*) or anti-LAMP1 (*red*) followed by nuclei counterstaining with Hoechst 33258 (*blue*). Merged images are shown to the *right*. Scale bar represents 10 μm. *G*, quantification of puncta colocalized with LC3 and LAMP1 was performed. Data are shown as the mean ± S.D of three independent experiments. NS, not significant. *H*, MN9D cells treated with or without 250 μM CuCl_2_ for 15 h were stained with Magic Red cathepsin B (Cat B). Nuclei were counterstained with Hoechst 33258 (*blue*). Merged images are shown to the *right*. Scale bar represents 20 μm. *I*, the relative intensity of each fluorescent signal of Cat B was quantified using ImageJ software. Data are shown as the mean ± S.D of three independent experiments. ∗∗∗*p* < 0.001. *J*, MN9D cells treated with or without 250 μM CuCl_2_ for 15 h and probed with LysoTracker Red. Representative confocal images are provided. Nuclei were counterstained with Hoechst 33258 (*blue*). Merged images are shown to the *right*. Scale bar represents 20 μm. *K*, the relative intensity of each fluorescent signal of LysoTracker Red was quantified using ImageJ software. Data are shown as the mean ± S.D of three independent experiments. ∗*p* < 0.05.

To determine the cause of impaired autophagic flux by CuCl_2_, we first examined whether the number or function of lysosomes or both was affected. Immunofluorescence analyses demonstrated that puncta distribution of the lysosome-associated membrane protein 1 (LAMP1) remained the same regardless of treatment (Fig. 4, *A* and *B*). Immunoblot analyses also indicated that levels of LAMP1 were unaltered by CuCl_2_ treatment (Fig. 4, *C* and *D*), implying that the CuCl_2_-induced impairment in autophagic flux is likely not due to reduced numbers of lysosomes. We next determined the effect of CuCl_2_ treatment on the rate of autophagosome-lysosome fusion by analyzing the co-localization of LC3 and LAMP1 following treatment with CuCl_2_ and Torin-1, an inhibitor of mTOR. No discernible change in the degree of co-localization between LC3 and LAMP1 was detected (Fig. 4, *F* and *G*), indicating that CuCl_2_ did not affect the rate of fusion between autophagosomes and lysosomes. Based on these data, we hypothesized that CuCl_2_-induced impairment of autophagic flux may be ascribed to a loss of lysosomal function. The optimal function of lysosomal hydrolases requires lysosomes to maintain a low internal pH (41). Therefore, we tried to determine the hydrolase catalytic activity and luminal pH. Immunoblot analyses indicated that the cleaved form of cathepsin D (c-CatD) decreased following CuCl_2_ treatment (Fig. 4, *C* and *E*). Similarly, fluorometric assay and quantitative analyses indicated that cathepsin B activity decreased following drug treatment (Fig. 4, *H* and *I*). To check whether the luminal pH of lysosomes was altered by CuCl_2_, cells were stained with LysoTracker Red, a fluorescent dye that labels acidic organelles. In comparison with non-treated cells, CuCl_2_-treated cells lost fluorescence (Fig. 4, *J* and *K*). These data support the notion that the luminal acidity of lysosomes was affected by CuCl_2_ and the resultant disruption of proteolytic activity seems to be associated with impaired lysosomal activity.

### CuCl_2_-induced surge of cytosolic Ca^2+^ is responsible for impaired autophagic flux

**Figure 5:**
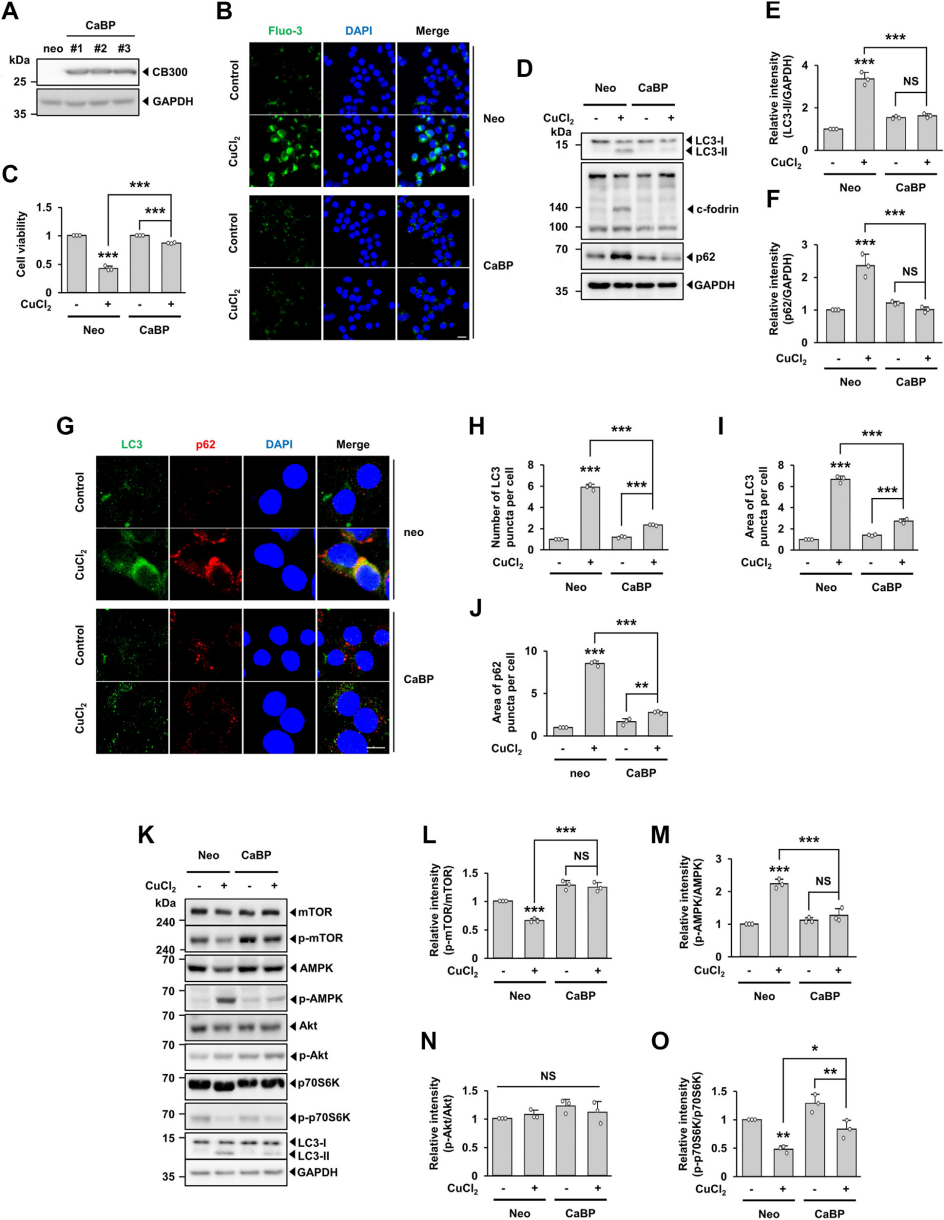
**Cytosolic Ca**^**2+**^**-dependent impairment of autophagy flux in MN9D cells following CuCl**_**2**_**treatment.***A*, immunoblot analyses were performed using anti-CB300 antibody using cell lysates obtained from stable MN9D/Neo and MN9D/CaBP cells. Anti-GAPDH antibody was utilized as a loading control. *B*–*O*, MN9D/Neo or MN9D/CaBP cells were treated with or without 250 μM CuCl_2_ for 15 h. *B*, cells were stained with 3 μM Fluo-3 (*green*). Representative confocal images are provided. Nuclei were counterstained with Hoechst 33258 (*blue*). Merged images are shown to the *right*. Scale bar represents 20 μm. *C*, MTT reduction assay was performed to assess cell viability, which was expressed as a percentage over untreated matching control (value = 1). Data are shown as the mean ± S.D. of three independent experiments. Two-way ANOVA followed by Tukey’s post hoc test was performed. ∗∗∗*p* < 0.001. *D*, immunoblot analyses were performed using anti-LC3, anti-fodrin, or anti-p62 antibodies. The calpain-cleaved band of fodrin (c-fodrin) was detected by an anti-fodrin antibody. Relative intensities of LC3-II (*E*) and p62 (*F*) signals were measured using ImageJ software, normalized by the intensity of GAPDH signal, and expressed as fold change relative to untreated control (value = 1). Data are shown as the mean ± S.D of three independent experiments. Two-way ANOVA followed by Tukey’s post hoc test was performed. ∗∗∗*p* < 0.001; NS, not significant. *G*, immunocytochemical analyses were performed using anti-LC3 (*green*) and anti-p62 (*red*), and nuclei were counterstained with Hoechst 33258 (*blue*). Representative confocal images are provided. Merged images are shown to the *right*. Scale bar represents 10 μm. The number (*H*) and area (*I*) of LC3 and area of p62 (*J*) puncta per cell were quantified using ImageJ software. Data are shown as the mean ± S.D of three independent experiments. Two-way ANOVA followed by Tukey’s post hoc test was performed. ∗∗*p* < 0.01; ∗∗∗*p* < 0.001. *K*, cellular lysates were subjected to immunoblot analyses using the indicated antibodies. After normalization against the intensity of total protein, relative intensities of the phosphorylated forms of mTOR (*L*), p-AMPK (*M*), p-Akt (*N*), and p-p70S6K (*O*) are expressed as fold change relative to untreated control (value = 1). Data are shown as the mean ± S.D of three independent experiments. Two-way ANOVA followed by Tukey’s post hoc test was performed. ((*N*) ANOVA *p* value is 0.229215) ∗*p* < 0.05; ∗∗*p* < 0.01; ∗∗∗*p* < 0.001; NS, not significant.

A previous study demonstrates that cytosolic Ca^2+^ levels increase in MCF7 cells following treatment with CuCl_2_ (42). Therefore, we investigated whether CuCl_2_ treatment induces a surge in cytosolic Ca^2+^ and dysregulates autophagy in MN9D cells. We first checked cytosolic Ca^2+^ levels using Fluo-3. Confocal microscopy showed that Fluo-3 intensity in cells increased when treated with CuCl_2_ (Fig. S3, *A* and *B*). Furthermore, we found that Ca^2+^-activated cleavage of fodrin (c-fodrin) was increased (Fig. S3*C*), indicating a CuCl_2_-induced surge of cytosolic Ca^2+^ in MN9D cells. Based on previous reports demonstrating that calbindin-D28K, the large EF-hand family of Ca^2+^ binding proteins, has neuroprotective effects in several neurodegenerative disease models (43, 44), we determined whether buffering of the CuCl_2_-induced surge of cytosolic Ca^2+^ affects the rate of cell death and accompanying impairment in autophagy. For this purpose, we used MN9D cells stably overexpressing calbindin-D28K (MN9D/CaBP cells) or vector alone (MN9D/Neo cells). Among three independent MN9D/CaBP cell lines established (#19, 24 and 26), we routinely used #19 cell line for the study (Fig. 5*A*). Fluo-3 images indicated that the CuCl_2_-induced surge of cytosolic Ca^2+^ was blocked in MN9D/CaBP cells (Fig. 5*B*). MTT reduction assay showed that the CuCl_2_-induced cell death was blocked in MN9D/CaBP cells (Fig. 5*C*). To compare the extent of autophagy, we determined levels of LC3-II and p62 in both types of cells. In MN9D/CaBP cells, the CuCl_2_-induced appearance of LC3-II was blocked (Fig. 5, *D* and *E*). Similarly, the CuCl_2_-induced accumulation of p62 was attenuated in MN9D/CaBP cells (Fig. 5, *D* and *F*). In this condition, Ca^2+^-mediated activation of calpain cleaved fodrin to generate c-fodrin in MN9D/Neo cells and this event was blocked in MN9D/CaBP cells. Consistently, comparative immunofluorescence analyses showed that the CuCl_2_-induced increase in the number and area of LC3 puncta and area of p62 puncta was attenuated in MN9D/CaBP cells (Fig. 5, *G*–*J*). A similar pattern of blockade was observed in MN9D cells co-treated with BAPTA-AM (BAPTA), a cell-permeable selective Ca^2+^ chelator. Consequently, immunoblot analyses indicated that the appearance of LC3-II and accumulation of p62 was blocked in cells co-treated with CuCl_2_ and BAPTA (Fig. S4, *A*–*C*). Increased appearance of LC3 puncta was attenuated in cells co-treated with CuCl_2_ and BAPTA (Fig. S4*D*). CuCl_2_-induced disappearance of LysoTracker Red fluorescence was restored in cells co-treated with BAPTA (Fig. S4*E*), further supporting a notion that drug-induced surge of cytosolic Ca^2+^ is responsible for causing impaired autophagy flux. To delineate the underlying mechanism of autophagy signal, we examined mTOR-related signaling pathways. As expected, the decreased levels of p-mTOR and p-p70S6K and simultaneously increased levels of p-AMPK in MN9D/Neo cells were largely reversed in MN9D/CaBP cells (Fig. 5, *K*–*O*).

**Figure 6:**
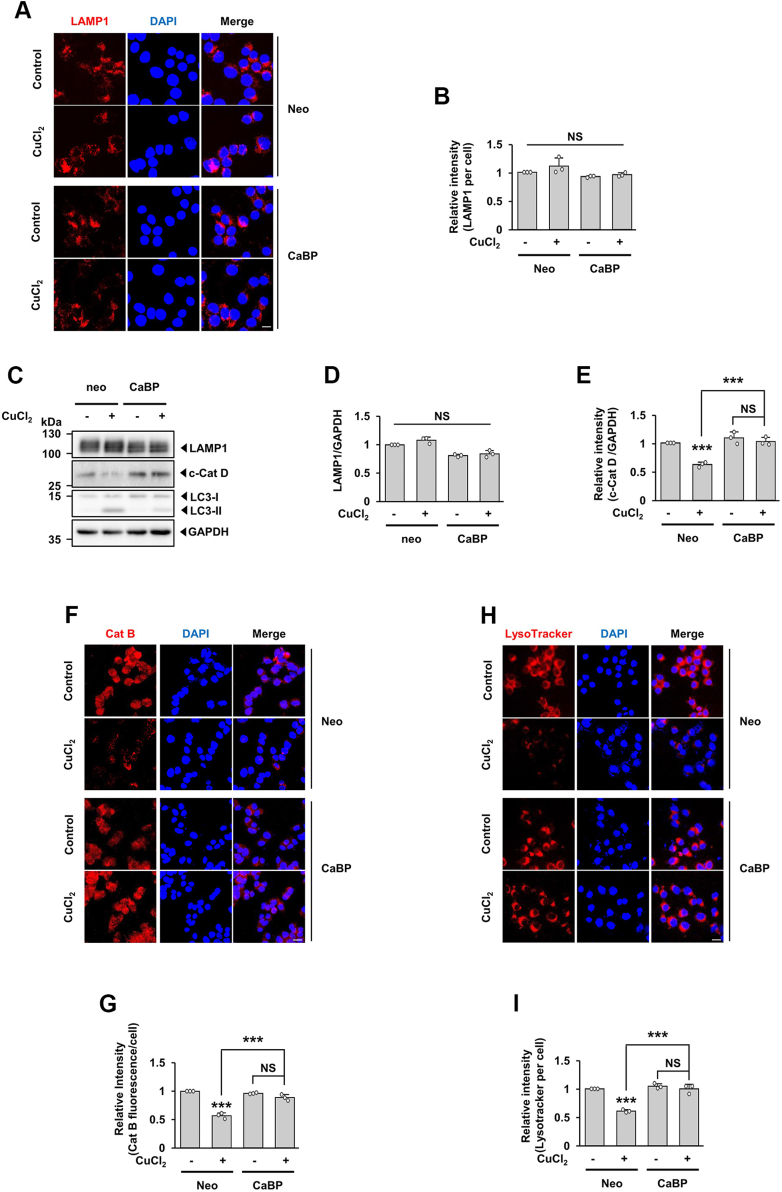
**Rescue from CuCl**_**2**_**-induced dysregulated lysosomal function *via* buffering cytosolic Ca**^**2+**^**.** MN9D/Neo or MN9D/CaBP cells were treated with or without 250 μM CuCl_2_ for 15 h. *A*, immunocytochemical analyses were performed using anti-LAMP1 (*red*) and nuclei were counterstained with Hoechst 33258 (*blue*). Representative confocal images are provided. Merged images are shown to the *right*. Scale bar represents 20 μm. *B*, relative intensity of LAMP1 per cell was quantified using ImageJ software. Data are shown as the mean ± S.D. of three independent experiments. Two-way ANOVA was performed (*p* value is 0.401729). NS, not significant. *C*, cellular lysates were subjected to immunoblot analyses using the indicated antibodies. Relative intensities of LAMP1 (*D*) and c-Cat D (*E*) signals were measured using ImageJ software, normalized by the intensity of GAPDH signal, and expressed as fold change relative to untreated control (value = 1). Data are shown as the mean ± S.D of three independent experiments. Two-way ANOVA followed by Tukey’s post hoc test was performed ((*D*) ANOVA *p* value is 0.318773). ∗∗∗*p* < 0.001; NS, not significant. *F*, after CuCl_2_ treatment, cells were stained with Magic Red Cat B. Nuclei were counterstained with Hoechst 33258 (*blue*). Merged images are shown to the *right*. Representative confocal images are provided. Scale bar represents 20 μm. *G*, the relative intensity of each fluorescent signal of Cat B per cell was quantified using ImageJ software. Data are shown as the mean ± S.D. of three independent experiments. Two-way ANOVA followed by Tukey’s post hoc test was performed. ∗∗∗*p* < 0.001; NS, not significant. *H*, after CuCl_2_ treatment, cells were stained with LysoTracker Red. Nuclei were counterstained with Hoechst 33258 (*blue*). Merged images are shown to the *right*. Representative confocal images are provided. Scale bar represents 20 μm. *I*, the relative intensity of each fluorescent signal of LysoTracker Red per cell was quantified using ImageJ software. Data are shown as the mean ± S.D of three independent experiments. Two-way ANOVA followed by Tukey’s post hoc test was performed. ∗∗∗*p* < 0.001; NS, not significant.

To test whether the buffering of cytosolic Ca^2+^ in CuCl_2_-treated MN9D cells affects the number of lysosomes or blocks the loss of lysosomal activity, we first compared expression levels of LAMP1 in MN9D/Neo cells and MN9D/CaBP cells. As determined by immunofluorescent staining, levels of LAMP1 were not altered in either cell type regardless of CuCl_2_ treatment (Fig. 6, *A* and *B*). Similarly, immunoblot analyses indicated no discernible alteration of LAMP1 levels (Fig. 6, *C* and *D*). However, fluorogenic activity assay and quantitative analyses indicated that the CuCl_2_-induced decrease in fluorescence intensity of cathepsin B activity (Fig. 6, *F* and *G*) and LysoTracker Red (Fig. 6, *H* and *I*) was blocked in MN9D/CaBP cells. Consistently, immunoblot analyses showed that drug-induced decreases in levels of the c-CatD were reversed in MN9D/CaBP cells (Fig. 6, *C* and *E*).

**Figure 7:**
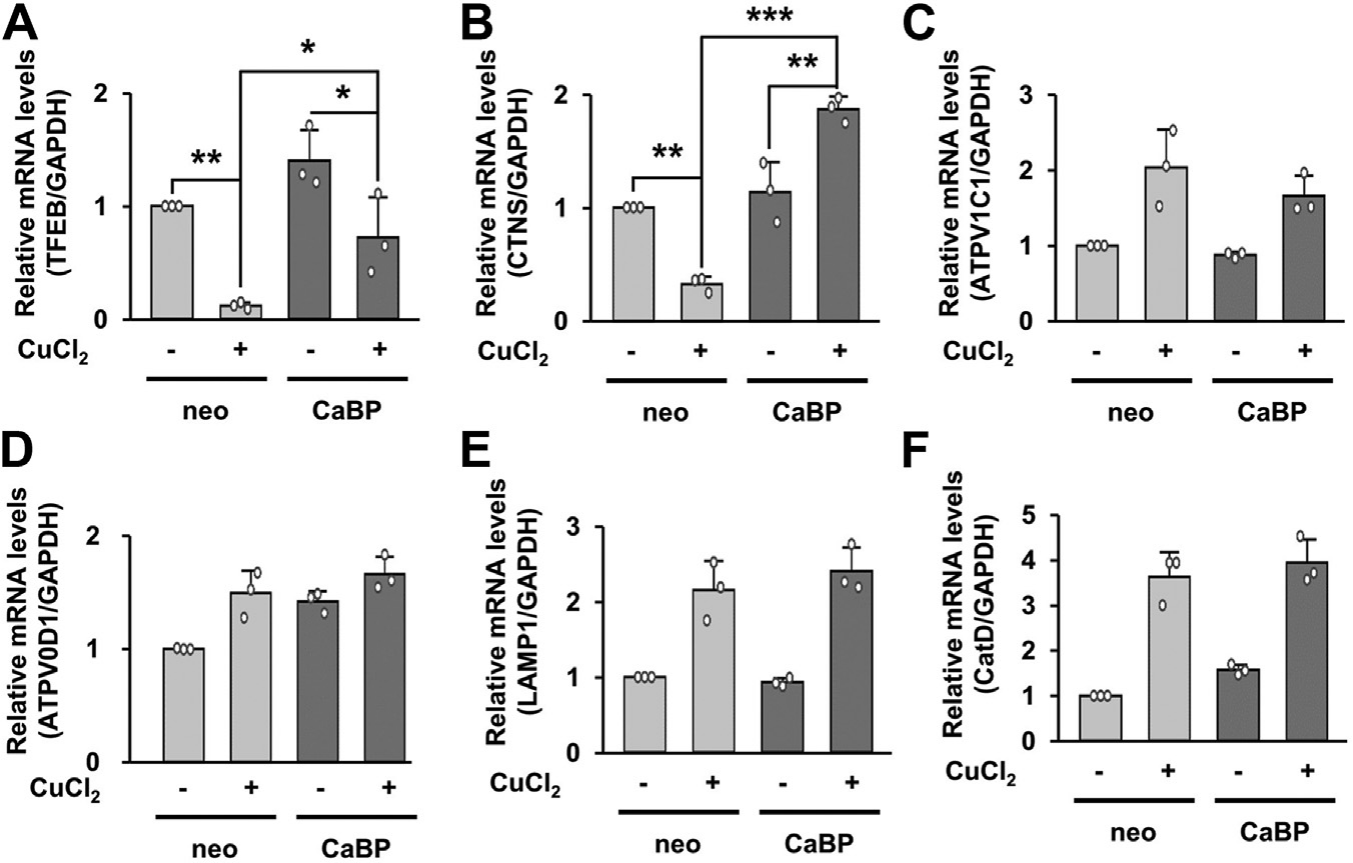
**Transcriptional levels of lysosome-related genes following CuCl**_**2**_**treatment.** Both MN9D/Neo and MN9D/CaBP cells were processed for real-time RT-PCR to analyze mRNA levels of analyzed TFEB (*A*), CTNS (*B*), ATPV1C1 (*C*), ATPV0D1 (*D*), LAMP1 (*E*), and CatD (*F*) after treatment with or without CuCl_2_ treatment for 15 h. mRNA levels of each gene were normalized by the levels of GAPDH mRNA and expressed as fold change relative to untreated control (value = 1). Some of the additive information is described in the Experimental procedures section. Data are shown as the mean ± S.D of three independent experiments. Two-way ANOVA followed by Tukey’s post hoc test was performed. ((*C*) two-way ANOVA *p* value is 0.461295, (*D*) two-way ANOVA *p* value is 0.145812, (*E*) two-way ANOVA *p* value is 0.302986, (*F*) two-way ANOVA *p* value is 0.561369). ∗*p* < 0.05; ∗∗*p* < 0.01; ∗∗∗*p* < 0.001.

To check the changes in the transcription rate of lysosome-related genes following CuCl_2_ treatment and how buffering of cytosolic Ca^2+^ affects these events, we performed real-time RT-PCR for six lysosome-related genes using MN9D/Neo and MN9D/CaBP cells following CuCl_2_ treatment. Levels of transcription factor EB mRNA (TFEB; a master gene for lysosomogenesis) were dramatically decreased in MN9D/Neo cells and these events were reversed in MN9D/CaBP cells after CuCl_2_ treatment (Fig. 7*A*). Levels of CTNS mRNA (a lysosomal H^+^ and cysteine transporter) were decreased in MN9D/Neo cells whereas its levels were enhanced in MN9D/CaBP cells after CuCl_2_ treatment (Fig. 7*B*). For the remaining genes, levels of mRNA were all increased in both MN9D/Neo and MN9D/CaBP cells upon CuCl_2_ treatment (Fig. 7, *C*–*F*). Interestingly, drug-induced alteration of vacuolar-type V-ATPase (V-ATPase) mRNA levels (ATPV1C1, ATPV0D1) was not significantly different in both cell lines (Fig. 7, *C* and *D*). Similarly, no discernible changes in transcriptional levels of LAMP1 and CatD were detected in both cell lines (Fig. 7, *E* and *F*). In considering no changes of LAMP1 protein levels following CuCl_2_ treatment (*e.g.*, Fig. 6, *C* and *D*), however, the reason for the reciprocal patterns of LAMP1 mRNA *versus* protein is unknown and needs further investigation in both MN9D/Neo and MN9D/CaBP cells following CuCl_2_ treatment.

Previous reports including ours demonstrate that metal ions can serve as pro-oxidants (45, 46, 47). When we measured DCF fluorescence as an indicator of ROS, we found that ROS was increased in MN9D cells treated with CuCl_2_ and was blocked in MN9D cells co-treated with an antioxidant, N-acetylcysteine (NAC) (Fig. S5*A*). However, both immunoblot and immunofluorescence analyses showed that the CuCl_2_-induced appearance of LC3-II and accumulation of p62 were not blocked in MN9D cells co-treated with NAC (Fig. S5, *B*–*H*). Furthermore, drug-induced loss of LysoTracker Red fluorescence (Fig. S5*I*) was not restored in NAC-co-treated cells. Accordingly, our data suggest that a surge in cytosolic Ca^2+^ but not ROS is associated with the CuCl_2_-induced impairment of autophagy flux in MN9D cells.

### Autophagic flux is impaired by CuCl_2_ treatment and recovered *via* buffering of cytosolic Ca_2_^+^ levels in primary cultures of mouse cortical neuron

**Figure 8:**
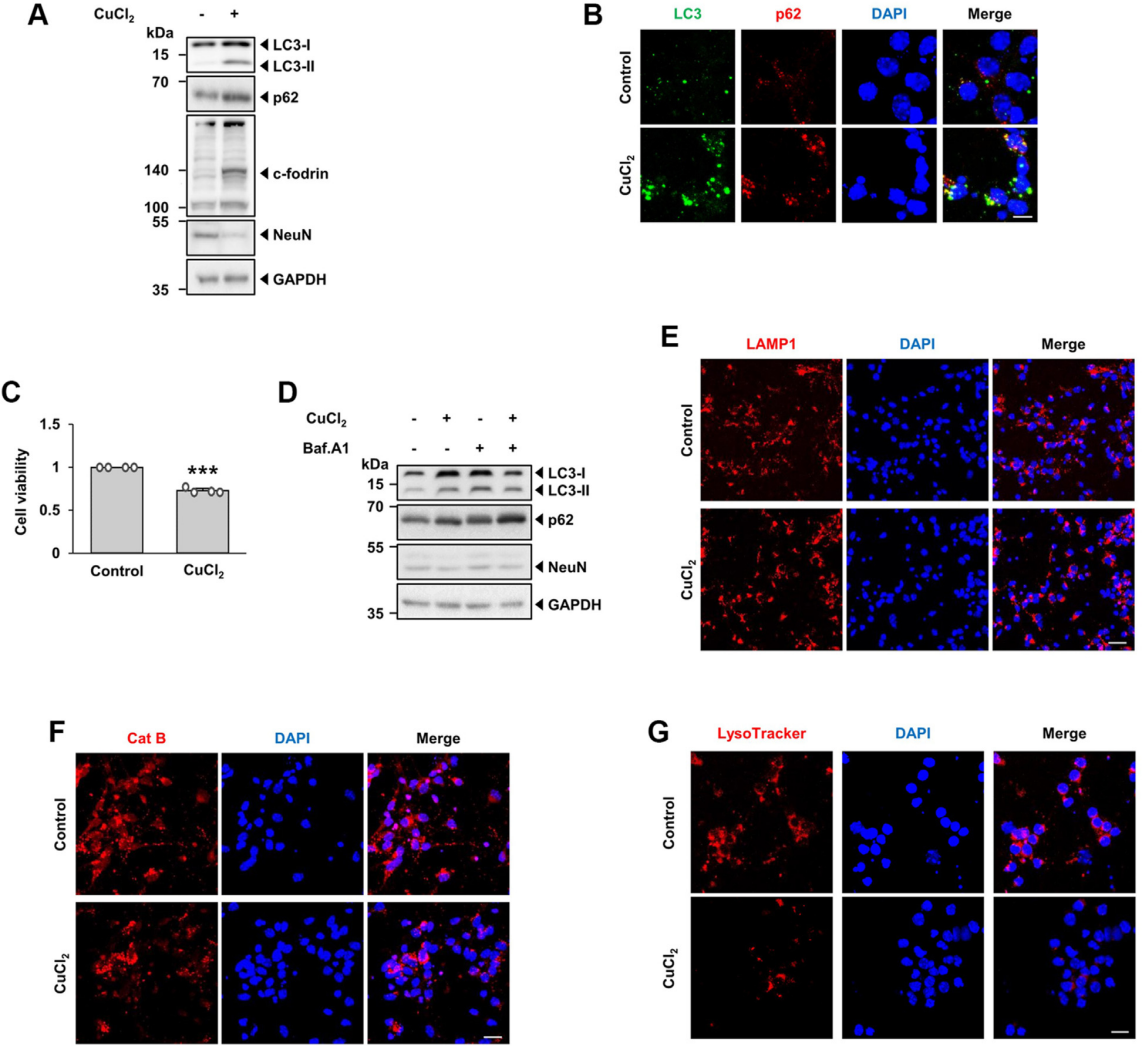
**Autophagic flux impairment and lysosomal dysfunction induced by CuCl**_**2**_**treatment in cortical neuronal cells.** Primary cultures of cortical neurons established as described in Experimental Procedures were treated with or without 250 μM CuCl_2_ for 18 h. *A*, immunoblot analyses were performed using the indicated antibodies. *B*, immunocytochemical localization analysis was performed using anti-LC3 (*green*) and anti-p62 (*red*) antibodies followed by counterstaining with Hoechst 33258 (*blue*). Merged images are shown to the *right*. Representative confocal images are provided. Scale bar represents 10 μm. *C*, MTT reduction assay was performed to assess cell viability, which was expressed as a fold change relative to untreated control cell (value = 1). Data are shown as the mean ± S.D of four independent experiments. ∗∗∗*p* < 0.001. *D*, immunoblot analyses were performed using the indicated antibodies in the presence or absence of 50 nM Baf.A1 for the final 4 h. *E*, immunocytochemical analyses were performed using anti-LAMP1 (*red*) followed by nuclei counterstaining with Hoechst 33258 (*blue*). Merged images are shown to the *right*. Scale bar represents 20 μm. *F* and *G*, after CuCl_2_ treatment, cells were stained with (*F*) Magic Red cathepsin B (Cat B) or (*G*) LysoTracker Red. Nuclei were counterstained with Hoechst 33258 (*blue*). Merged images are shown to the *right*. Representative confocal images are provided. Scale bar represents 20 μm. All data represent of three independent experiments.

**Figure 9:**
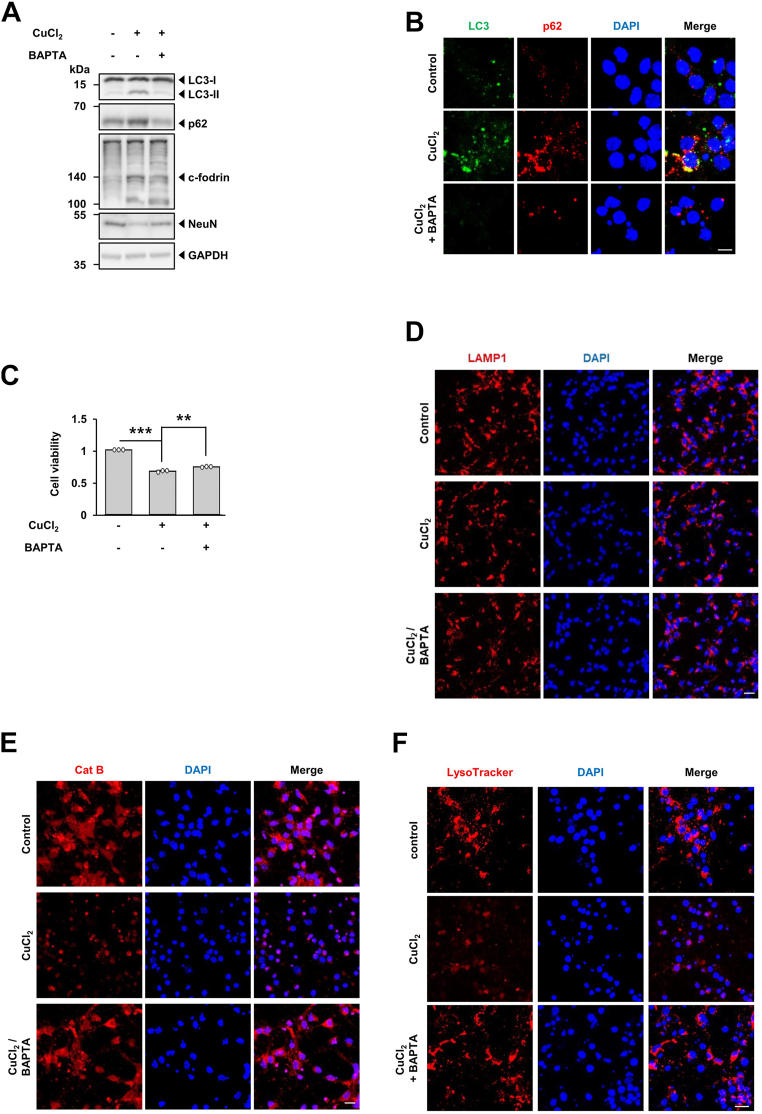
**Rescue of CuCl**_**2**_**-induced impairment of autophagic flux by buffering of drug-induced surge of cytosolic Ca**^**2+**^**in cortical neuronal cells.** Cortical neuronal cells were incubated with 250 μM CuCl_2_ for 15 h in the presence or absence of 40 μM BAPTA-AM. *A*, immunoblot analyses were performed using the indicated antibodies. *B*, immunocytochemical localization analysis was performed using anti-LC3 (*green*) and anti-p62 (*red*) antibodies followed by counterstaining with Hoechst 33258 (*blue*). Merged images are shown to the *right*. Representative confocal images are provided. Scale bar represents 10 μm. *C*, MTT reduction assay was performed to assess cell viability, which was expressed as a percentage over untreated control cells (value = 1). Data are shown as the mean ± S.D of three independent experiments. ∗∗*p* < 0.01; ∗∗∗*p* < 0.001. *D*, immunocytochemical analyses were performed using anti-LAMP1 (*red*) followed by nuclei counterstaining with Hoechst 33258 (*blue*). Merged images are shown to the *right*. Scale bar represents 20 μm. *E* and *F*, after CuCl_2_ treatment, cells were stained with (*E*) Magic Red cathepsin B (Cat B) or (*F*) LysoTracker Red. Nuclei were counterstained with Hoechst 33258 (*blue*). Merged images are shown to the *right*. Scale bar represents 20 μm. All data represent of three independent experiments.

After we set up the sequence of impaired autophagy flux in MN9D cells following CuCl_2_ treatment, we attempted to confirm these findings using primary cultures of cortical neuron upon exposure to CuCl_2._ Immunoblot analyses indicated that levels of LC3-II and p62 were increased (Fig. 8*A*). In concomitant with these events, levels of the calpain-mediated c-fodrin were enhanced following drug treatment, suggesting drug-induced surge of cytosolic Ca^2+^ may trigger these changes. Levels of the NeuN, a neuronal nuclear antigen was decreased upon exposure to CuCl_2_. Immunocytochemistry analyses indicated that extent of LC3-II and p62 puncta were increased following CuCl_2_ treatment (Fig. 8*B*). In this condition, MTT assay showed that cell viability was decreased upon CuCl_2_ treatment (Fig. 8*C*). To check autophagic flux after CuCl_2_ treatment, we then performed immunoblot analyses in the presence or the absence of Baf.A1. Immunoblot analyses indicated that co-treatment with CuCl_2_ and Baf.A1 did not further enhance levels of LC3-II and p62 as compared with levels in cortical neurons treated only with CuCl_2_ (Fig. 8*D*), suggesting that CuCl_2_-induced accumulation of LC3-II and p62 in cortical neurons was due to a blockade of autophagic flux at the lysosomal level. Immunocytochemistry showed that levels of LAMP1 was similar regardless of CuCl_2_ treatment (Fig. 8*E*). However, fluorogenic assay showed that the intensity of Cathepsin B activity and LysoTracker Red was decreased upon exposure of cortical neurons to CuCl_2_ treatment (Fig. 8, *F* and *G*), suggesting that the luminal acidity but not number of lysosomes was affected by CuCl_2_. These patterns were quite similar to those observed in MN9D cells. To confirm whether these events are affected by CuCl_2_-induced surge of cytosolic Ca^2+^, cortical neurons were co-treated with to without BAPTA. Immunoblot analyses showed that co-treatment with BAPTA attenuated CuCl_2_-induced increase in LC3-II and p62 whereas drug-induced decrease in NeuN levels was restored (Fig. 9*A*). Similarly, immunocytochemistry indicated that CuCl_2_-induced increase in LC3 and p62 puncta was reversed after co-treatment with BAPTA (Fig. 9*B*). The MTT reduction assay indicated that cell viability was increased in cortical neurons treated with CuCl_2_ and BAPTA co-treatment as compared to cells treated only with CuCl_2_ (Fig. 9*C*). As expected, immunofluorescence analyses demonstrated that puncta distribution of LAMP1 remained the same in all groups regardless of treatment (Fig. 9*D*). However, fluorogenic activity assay indicated that the CuCl_2_-induced decrease in fluorescence intensity of cathepsin B activity (Fig. 9*E*) and LysoTracker Red (Fig. 9*F*) was blocked in cortical neurons co-treated with BAPTA, largely confirming the findings in MN9D cells.

## Discussion

In the present study, we attempted to establish our notion that a CuCl_2_-induced surge of cytosolic Ca^2+^ is critically involved in dysregulated autophagy in neuronal cells. Results from cells overexpressing calbindin-D28K or cells co-treated with BAPTA demonstrated that the CuCl_2_-induced surge of cytosolic Ca^2+^ leads to insufficient autophagic flux by causing lysosomal deficits in maintaining luminal acidic pH. Therefore, buffering the surge of cytosolic Ca^2+^ restores an optimal lysosomal luminal pH and consequently maintains a functional state of autophagic flux. Unlike the observation that NAC attenuates ROS-mediated dysregulated autophagic flux following 6-OHDA treatment (10), CuCl_2_-induced dysregulated autophagic flux was not blocked in the presence of NAC. Although we did not demonstrate whether CuCl_2_ dysregulates the autophagy-lysosome pathway at different levels, multiple unrelated assays clearly demonstrate that lysosomes under excessive cytosolic Ca^2+^ pressure fail to maintain the acidic pH and a normal physiological function. This argument is consistent with previous findings, including those from our laboratory, that dysregulation of the autophagy-lysosome pathway at various levels comprises a critical step in the progression of neurodegeneration (48, 49, 50, 51).

Accumulating evidence indicates that disruption of cytosolic Ca^2+^ homeostasis is related to neurodegenerative diseases (52, 53, 54). Since the Ca^2+^ hypothesis in AD was first proposed (55), it has been accepted that perturbed Ca^2+^ homeostasis is implicated in age-related cognitive impairment and the development of AD (54, 56). For instance, Ca^2+^ influx through the plasma membrane, and/or release from the endoplasmic reticulum and mitochondria contributes to perturbed Ca^2+^ homeostasis (57, 58, 59). A surge of cytosolic Ca^2+^ destroys the balance of Ca^2+^-dependent calcineurin and Ca^2+^/calmodulin-dependent protein kinase II (CaMKII) activity. This imbalance leads to instability of synaptic connections and the late onset of synaptic degeneration (60). We also report that buffering of cytosolic Ca^2+^ blocks neuronal death in experimental models of PD (48, 61). Similarly, increased cytosolic Ca^2+^ in motor neurons is also linked to the development of ALS (62, 63). Recently, an interesting relationship between proteins coded by ALS-related genes and Ca^2+^ accumulation was proposed (64). For instance, excessive cytosolic Ca^2+^ in the early stage is attributed to cleavage or degradation of ALS-related proteins. These events are mediated by proteolytic activation of calpain and caspases as well as by activation of autophagy. Indeed, it has been shown that Ca^2+^ plays an important role in the autophagy-lysosomal pathway (65). For example, Ca^2+^-dependent kinases can either activate or inhibit the autophagy signaling pathway (66, 67). During acute axonal degeneration in the optic nerve, increases in autophagosome formation and intra-axonal Ca^2+^ levels are detected (68). Application of a Ca^2+^ channel blocker attenuates axonal degeneration, whereas application of Ca^2+^ ionophores aggravates the degenerative phenotype, suggesting that increased postlesional autophagy is Ca^2+^-dependent. These studies support our findings that the CuCl_2_-induced surge of Ca^2+^ dysregulates autophagic flux and that buffering cytosolic Ca^2+^ restores autophagic flux *via* the autophagy-lysosome pathway.

CTNS is the gene that encodes the protein cystinosin. Cystinosin is a lysosomal seven-transmembrane protein that functions as a symporter transporting protons and cysteine out of the lysosome (69). Mutations of CTNS cause lysosomal storage disease and defect of cystinosin leads to surge of ROS, and mitochondrial and lysosomal dysfunction (70, 71). Our real-time RT-PCR data indicated that levels of CTNS mRNA were remarkably decreased when MN9D/Neo cells were treated with CuCl_2._ However, we found levels of CTNS mRNA enhanced over the untreated control levels in MN9D/CaBP cells following CuCl_2_ treatment (Fig. 7*B*). Although we do not determine the functional consequence of these changes, however, we are tempted to postulate that CuCl_2_-induced decrease in CTNS mRNA leads to the dysfunctional lysosome and that buffering of calcium can contribute to restoring lysosomal function. The lysosomal function is regulated by a variety of factors such as lysosomal luminal pH, proteases, and ion channels (72). Most lysosomal membrane proteins and enzymes are synthesized in the rough endoplasmic reticulum and are recruited to lysosomal compartments in small vesicles (73, 74). Their synthesis is controlled by TFEB, which promotes the transcription of nuclear genes and thereby acts as a master regulator of lysosome biogenesis (75). Our results demonstrating that CuCl_2_-induced decrease in TFEB mRNA levels are significantly blocked in MN9D/CaBP cells imply that buffering of cytosolic Ca^2+^ play a critical role in preserving lysosomal function in cells. It is also intriguing to investigate why transcriptional levels of some of lysosomal genes increase in CaBP-expressing cell lines. It may be ascribed to the fact that Ca^2+^ is well-known to regulate transcription at all steps (76). Its critical involvement in gene transcription has been observed in several biological events including dendritic growth, neuronal plasticity and development of synapse in neuron (77). Similarly, several studies have shown that copper also plays an important role in transcriptional mechanism. For example, Cu ions are required to activate MEK/ERK pathway and increased expression of various transcription factors (78). Cu^2+^ and copper nanoparticles affect neuronal repair-gene transcription levels in olfactory mucosa (79). Cu ions bind classic metal-binding transcription factors and chaperones, including MTF1 and SP1, and regulate gene expression (80). With this in mind, we are further tempting to postulate that there may be synergic interplay between calcium and copper as well.

Several parameters have been used to evaluate lysosomal functions (81, 82). For example, a lysosomal function can be reliably achieved by measuring lysosomal pH and degradation activity. This is because a characteristic feature of lysosomes, and one with physiological relevance to their degradative function, is a highly acidic pH (∼4.5–5.5). Using both lysosomotropic fluorogenic staining methods and immunoblot analyses, we demonstrated that CuCl_2_ causes a failure in maintaining acidic lysosome luminal pH. We also found that these phenomena can be reversed by buffering the CuCl_2_-induced surge of cytosolic Ca^2+^ *via* overexpression of calbindin-D28K or co-treatment with BAPTA. These results are in line with our previous findings that a neurotoxin-induced surge in cytosolic Ca^2+^ suppresses autophagic degradation *via* raising lysosomal pH and that reducing cytosolic Ca^2+^ restores lysosomal luminal pH and normal function (48). Altogether, these results indicate that functional lysosomes are required for cell protection against CuCl_2_-mediated neurotoxicity. We do not know how the CuCl_2_-induced surge of cytosolic Ca^2+^ leads to the loss of acidic luminal pH of lysosomes. Several lysosomal membrane proteins that regulate luminal acidity and ion homeostasis of lysosomes might be involved. V-ATPases are membrane proteins that couple the energy of ATP hydrolysis to proton transport and primarily function to acidify intracellular compartments (83). Unlike our expectation, we observe increase in V-ATPase mRNA levels in both MN9D/Neo and MN9D/CaBP cells following CuCl_2_ treatment. Any discernible statistical difference is detected (Fig. 7, *C* and *D*). These results do not rule out the possibility that dysregulated activity of V-ATPase is involved and, therefore, remains to be thoroughly determined. Considering that effective lysosomal acidification requires not only V-ATPase activity but also counter-ion flows (84), it would be necessary to thoroughly investigate whether the activity of counter-ions in facilitating lysosomal acidification is affected during CuCl_2_-mediated neurotoxicity.

Previously, lysosomal dysfunction was found to disrupt cellular homeostasis and is linked to neurodegenerative diseases. Genetic mutations in PD, frontotemporal dementia, and ALS are associated with lysosomal dysfunction, resulting in protein aggregation and dysregulation of autophagy and vesicular trafficking (85). An AD-related risk gene is also associated with lysosomes, and dysregulation of endo-lysosomal flux promotes AD pathology (86). Although we do not investigate which subsets of lysosomal genes are affected by these phenomena, our observations raise the possibility that a surge of cytosolic Ca^2+^ plays a critical role in manifesting involvement of the lysosomal pathway with the progression of neurodegeneration *via* regulation of TFEB activity. In conclusion, growing evidence implicates a role of lysosomal dysfunction in neurotoxicity and neurodegenerative diseases (69, 87). Therefore, we believe that various lysosomal pathways and their related components represent potential pharmacological targets. In particular, maintaining lysosomal homeostasis may be a therapeutic target of immediate focus.

## Experimental procedures

### Cell culture and drug treatment

The MN9D neuronal cell line, established by somatic fusion between embryonic mesencephalic neurons and N18TG neuroblastoma (88, 89), was cultured as previously described (90, 91). Briefly, MN9D cells were grown at 37°C in Dulbecco’s modified Eagle’s medium (DMEM; Sigma-Aldrich, D5648) supplemented with heat-inactivated 10% fetal bovine serum (Gibco, 26,140–079) on culture dishes coated with 25 μg/ml poly-D-lysine (Sigma-Aldrich, P0899) in an atmosphere of 90% room air and 10% CO_2_. For drug treatment, culture medium was changed to N2 serum-free defined medium with or without the indicated drugs followed by further incubation for the indicated time periods. Drugs used included CuCl_2_ (Sigma-Aldrich, C6917), Bafilomycin A1 (Baf.A1; Sigma-Aldrich, B1793), Torin-1 (Cell Signaling Technology, 14,379), BAPTA-AM (Invitrogen, B6769), and N-acetylcysteine (NAC; Sigma-Aldrich, A9165). Controls were not treated with any of these drugs at the matching time periods. Previously established MN9D stable cell lines overexpressing vector-only (MN9D/Neo cells) or calbindin-D28K (MN9D/CaBP cells) (61) were maintained in a culture medium containing 250 μg/ml G418 (A G. Scientific, G1033) and used for experiments. For the primary culture of cortical neurons, mouse cerebral cortices were acquired from E14.5 (Orient) and processed as previously described (92). Briefly, dissociated cortical neurons were plated on culture dishes coated with 100 μg/ml poly-D-lysine and 1 μg/ml laminin (Invitrogen, 23,017–015), and cultivated at 37 °C in an atmosphere of 95% room air/5% CO_2_ in minimum essential medium (MEM; Gibco, 21,103–049) supplemented with 1 mM sodium pyruvate (Sigma-Aldrich, P5280), 2 mM L-glutamine (Sigma-Aldrich, G8540), 0.6% glucose (Gibco, 15,023–021), penicillin-streptomycin (100 U/ml) and 10% FBS. After 24 h, the culture medium was changed to Neurobasal medium (Invitrogen, 21,103,049) supplemented with 2% B-27 (Gibco, 17,504,044), 0.5 mM L-glutamine, and 10 μM cytosine β-D-arabinofuranoside (Ara-C; Sigma-Aldrich, C1768). At 4 days *in vitro* (DIV4), neurons were treated with the empirically determined concentrations of the indicated drugs. All experimental procedures were approved by the Institutional Animal Care and Use Committee of Yonsei University (permissions IACUC (2018–01–689–03)).

### Transmission electron microscopy

Electron microscopy was performed as previously described (10). Briefly, MN9D cells grown in petri dishes were treated with 250 μM CuCl_2_ for 15 h followed by fixation with a mixture of 2% formaldehyde and 0.2% glutaraldehyde (Polysciences, Inc, 01,909) in 0.1 M cacodylate buffer (pH 7.2) for 30 min at 37 °C. To stop the fixation step, free aldehyde groups were blocked by soaking cells in 50 mM ammonium chloride in 0.1 M cacodylate buffer for 1 h. Cells were removed, sedimented by centrifugation, enclosed in liquefied 2% agarose, and post-fixed for 1 h with 1% osmium tetroxide (Electron Microscopy Sciences, EMS, 19,152). Subsequently, *en bloc* staining was performed with 1% aqueous uranyl acetate for 1 h. After staining, cells were dehydrated using an ethanol series and embedded in epoxy resin (Fluka, 45,345). Ultrathin sections (80 nm) were prepared, placed on Cu slot grids, stained with uranyl acetate and lead citrate, and observed at 80 kV with a Hitachi H-7650 electron microscope (Hitachi). Electron micrographs were taken with an 11-megapixel CCD XR611-M digital camera (Advanced Microscopy Techniques).

### Immunoblot analysis

At various time periods after drug treatment, MN9D cells were washed with cold phosphate-buffered saline (PBS; Lonza, 17–517Q) with 2 mM ethylenediaminetetraacetic acid (EDTA), lysed on ice in RIPA buffer (50 mM Tris-HCl [pH 7.4], 1% NP-40, 0.25% sodium deoxycholate, 150 mM NaCl, 1 mM EDTA, 0.1% sodium dodecyl sulfate (SDS)) containing complete protease inhibitor cocktail (Roche, 1,873,580), and subjected to homogenization by pipetting up and down using a 1-ml syringe with a 20-gauge needle. Cellular lysates were centrifuged at 13,000*g* for 20 min at 4 °C. Total collecting supernatant protein was quantified using 660 nm Protein Assay Reagent (Thermo Scientific, 22,660). Predetermined amounts of protein from each preparation were separated on 8 to 14% SDS polyacrylamide gel, transferred onto polyvinylidene fluoride (PVDF) membranes (Atto, AE-6667-P), and blocked with Tris-buffered saline containing 0.1% Tween-20 (TBST) and 5% skim milk for 30 min. PVDF membranes were incubated with the indicated primary antibodies overnight at 4 °C. The following primary antibodies were used: rabbit anti-LC3 antibody (Cell Signaling Technology, 2775), mouse anti-fodrin antibody (ENZO Life Sciences, BML-FG6090), guinea pig anti-p62/SQSTM1 antibody (Progen, GP62-C), rabbit anti-p-mTOR antibody (Cell Signaling Technology, 2971), rabbit anti-mTOR antibody (Cell Signaling Technology, 2972), rabbit anti-p-p70S6K antibody (Cell Signaling Technology, 2708), rabbit anti-p70S6K antibody (Cell Signaling Technology, 9234), rabbit anti-p-AMPK antibody (Cell Signaling Technology, 2535), rabbit anti-AMPK antibody (Cell Signaling Technology, 2603), rabbit anti-p-Akt antibody (Cell Signaling Technology, 4060), rabbit anti-Akt antibody (Cell Signaling Technology, 4685), rat anti-LAMP1 antibody (Developmental Studies Hybridoma Bank, 1D4B), rabbit anti-cathepsin D antibody (Santa Cruz, sc-10725), mouse anti-NeuN antibody (Sigma Aldrich, MAB377), mouse anti-Calbindin D28K antibody (Swant, 300) and rabbit anti-GAPDH antibody (Bethyl, A300–641A). After extensive washes with TBST, blots were incubated for 1 h at room temperature with horseradish peroxidase-conjugated anti-rabbit (Santa Cruz, sc-2004), anti-mouse (Santa Cruz, sc-2005), anti-guinea pig (Sigma-Aldrich, A5545), or anti-rat (Santa Cruz, sc-2006) secondary antibody. Specific bands on PVDF membranes were revealed upon the addition of a chemiluminescent substrate using an enhanced chemiluminescence kit (PerkinElmer Waltham, NEL105). Relative band intensity was measured using ImageJ software (National Institute of Health, Bethesda, MD, USA) and expressed as a value after normalization against the intensity of the GAPDH signal.

### Immunofluorescence staining

At various time periods after drug treatment, MN9D cells grown on glass coverslips were fixed with 4% paraformaldehyde (EMS, 15,170) at room temperature for 15 min and permeabilized with 0.1% saponin (Sigma, S4521) for 10 min. Coverslips were washed and incubated in PBS containing 0.2% Triton X-100 and 5% normal goat serum (Invitrogen, 16,210) for 1 h to block nonspecific sites. Subsequently, cells were incubated overnight at 4 °C with primary antibody in PBS containing 0.2% Triton X-100 and 1% normal goat serum. After washing with PBS, cells were incubated at room temperature for 1 h with appropriate secondary antibodies, which included Alexa 488-conjugated goat anti-rabbit IgG (Invitrogen, A11008), Alexa 568-conjugated goat anti-guinea pig IgG (Invitrogen, A11075), and Alexa 568-conjugated goat anti-rat IgG (Invitrogen, A11077). Nuclear counterstaining was carried out using the DNA helix intercalating dye Hoechst 33,258 (1 μg/ml; Molecular Probes, H-1398). Cells were mounted with Vectashield (Vector Laboratories, H1000). Z-stacked series of fluorescence images were acquired under a confocal microscope equipped with epifluorescence and a digital image analyzer (Zeiss, LSM 700). To quantify the punctate staining pattern of LC3, p62, and LAMP1 per cell, at least 30 cells were randomly selected from each of three independent experiments, Images acquired with 400× or 630× magnification were analyzed using ImageJ Imaging software. Quantification of co-localization was carried out to assess the regions of color overlay of two different fluorescence using ImageJ software. Diameters of 0.1 to 1.0 μm dot were included in data quantification. At least 30 cells were randomly selected from each of three independent experiments as previously described (89). Unless otherwise stated, the same primary antibodies were used for both immunoblot analysis and immunocytochemistry.

### Fluorescence imaging

Fluorescence intensity was quantified using ImageJ software. To measure intracellular Ca^2+^, MN9D cells treated with or without CuCl_2_ were stained with 3 μM Fluo-3 (Life Technology, F1242) mixed with pluronic acid (Life Technology, P3000MP) for 30 min at 37 °C. The intensity of the Fluo-3 signal was quantified from at least 100 randomly selected cells from each of three independent experiments. To explore the ectopic expression of a tandem mRFP-EGFP-LC3 probe, MN9D cells were transiently transfected using Lipofectamine2000 transfection reagent (Invitrogen, 11,668,019). Following drug treatment, at least 30 randomly selected cells from each of three independent experiments were subjected to quantitative analysis of the number of puncta that were EGFP^−^/mRFP^+^ or EGFP^+^/mRFP^+^. The Magic Red cathepsin B detection kit (ImmunoChemistry Technologies, 937) was used to measure cathepsin B activity. The intensity of Magic Red signal was quantified from at least 100 randomly selected cells from each of three independent experiments. To measure lysosomal activity after drug treatment, MN9D cells were loaded with 0.5 μM LysoTracker Red DND-99 (Invitrogen, L7528), a fluorescent probe highly selective for acidic organelles. The intensity of the LysoTracker Red signal was quantified from at least 50 randomly selected cells from each of three independent experiments. To measure intracellular ROS, MN9D cells treated with or without CuCl_2_ were stained with 3 μM CM-H2DCFDA (Invitrogen, C6827) for 30 min at 37 °C and washed twice with DMEM. The intensity of the CM-H2DCFDA signal was quantified from at least 100 randomly selected cells from each of three independent experiments.

### Cell viability assay

To examine cell viability after CuCl_2_ treatment, a colorimetric MTT reduction assay was performed. MN9D cells, MN9D/Neo cells, or MN9D/CaBP cells cultured on 24-well plates were incubated with 1 mg/ml MTT solution (Sigma-Aldrich M2128) at 37 °C for 1 h and lysed for 24 h in an extraction buffer containing 20% SDS in 50% aqueous dimethylformamide. The optical density of dissolved formazan grain was measured at 590 and 650 nm as test and reference wavelengths, respectively, by a VICTOR X5 Multilabel Plate Reader (PerkinElmer). Cell viability was expressed as a percentage relative to the value in untreated controls (100%).

### RNA extraction and real-time RT-PCR

Total RNA was isolated using TRI Reagent (Molecular Research Center Inc., TR118) and cDNA was transcribed using RevertAid First Strand cDNA Synthesis Kit (Thermo Scientific, K1622). Real-time PCR was performed using SYBR Premix Ex Taq II (Tli RNaseH Plus), ROX Plus (Takara, RR82LRB) according to the manufacturer’s protocol in 7300 Real-Time PCR system (Applied Biosystems). The thermal cycling conditions were as follows: 95 °C for 30 s, followed by 40 cycles at 95 °C for 5 sec and 60 °C for 31 sec. The expression level of the genes was calculated by the 2^−ΔΔCt^ ratio (93). Primer sequences used for qRT-PCR were described below. mRNA of GAPDH was used for normalization of an endogenous control. mTFEB forward 5′-AAGGTTCGGGAGTATCTGTCTG-3′, mTFEB reverse 5′-GGGTTGGAGCTGATATGTAGCA-3′ mCTNS forward 5′-ATGAGGAGGAATTGGCTGCTT-3′, mCTNS reverse 5′-ACGTTGGTTGAACTGCCATTTT-3′ mATPV1C1 forward 5′-ACTGAGTTCTGGCTCATATCTGC-3′, mATPV1C1 reverse 5′-TGGAAGAGACGGCAAGATTATTG-3′, mATPV0D1 forward 5′-ACAATGCCATTCTGGTGGA-3′, mATPV0D1 reverse 5′-ACATGGCATCAGCTGTGGT-3′, mLAMP1 forward 5′-GACCCTGAAAGTGGAGAACAA -3′, mLAMP1 reverse 5′-GGGCATCAGGAAGAGTCATATT-3′, mCatD forward 5′-CCTGGGCGATGTCTTCATT-3′, mCatD reverse 5′-GTGGAGAAGGAGCAAGTTAGAG-3′, mGAPDH forward 5′-CATGGCCTTCCGTGTTCCTA-3′, and mGAPDH reverse 5′-CCTGCTTCACCACCTTCTTG A-3′.

### Statistical analysis

Data are expressed as the mean ± standard deviation (SD) from three independent experiments. To determine the significance of differences between groups, two-tailed Student’s t-tests or two-way ANOVA followed by Tukey’s post hoc tests were performed using Matlab (The MathWorks, Inc., Natick, MA, USA) and Python (The Python Software Foundation, Wilmington, DE, USA). Values of ∗*p* < 0.05, ∗∗*p* < 0.01, and ∗∗∗*p* < 0.001 were considered statistically significant. NS stands for not statistically significant.

## Data availability

The data that support the findings of this study are available from the authors upon reasonable request.

## Supporting information

This article contains supporting information.

## Conflict of interest

The authors declare that they have no conflicts of interest with the contents of this article.
