# Supplementary material for: A surge of cytosolic calcium dysregulates lysosomal function and impairs autophagy flux during cupric chloride–induced neuronal death
Source: J Biol Chem. 2023 Nov 21;300(1):105479. doi: 10.1016/j.jbc.2023.105479 (PMC10750191; doi:10.1016/j.jbc.2023.105479)
Supplement: Supporting information [file mmc1.pdf]

A surge of cytosolic calcium dysregulates lysosomal function and impairs autophagy flux during cupric chloride-induced neuronal death

Yoonkyung Kim[1*], Yangsin Lee[2*], Minjung Choo[1], Nuri Yun[1,3], Jin Won Cho[1,2**],

and Young J. Oh[1,3**]

[1]Department of Systems Biology Yonsei University College of Life Science and Biotechnology; [2]Glycosylation Network Research Center, Yonsei University, Seoul 03722; [3]GNT Pharma Science Technology Center for Health, Incheon 21983, Korea

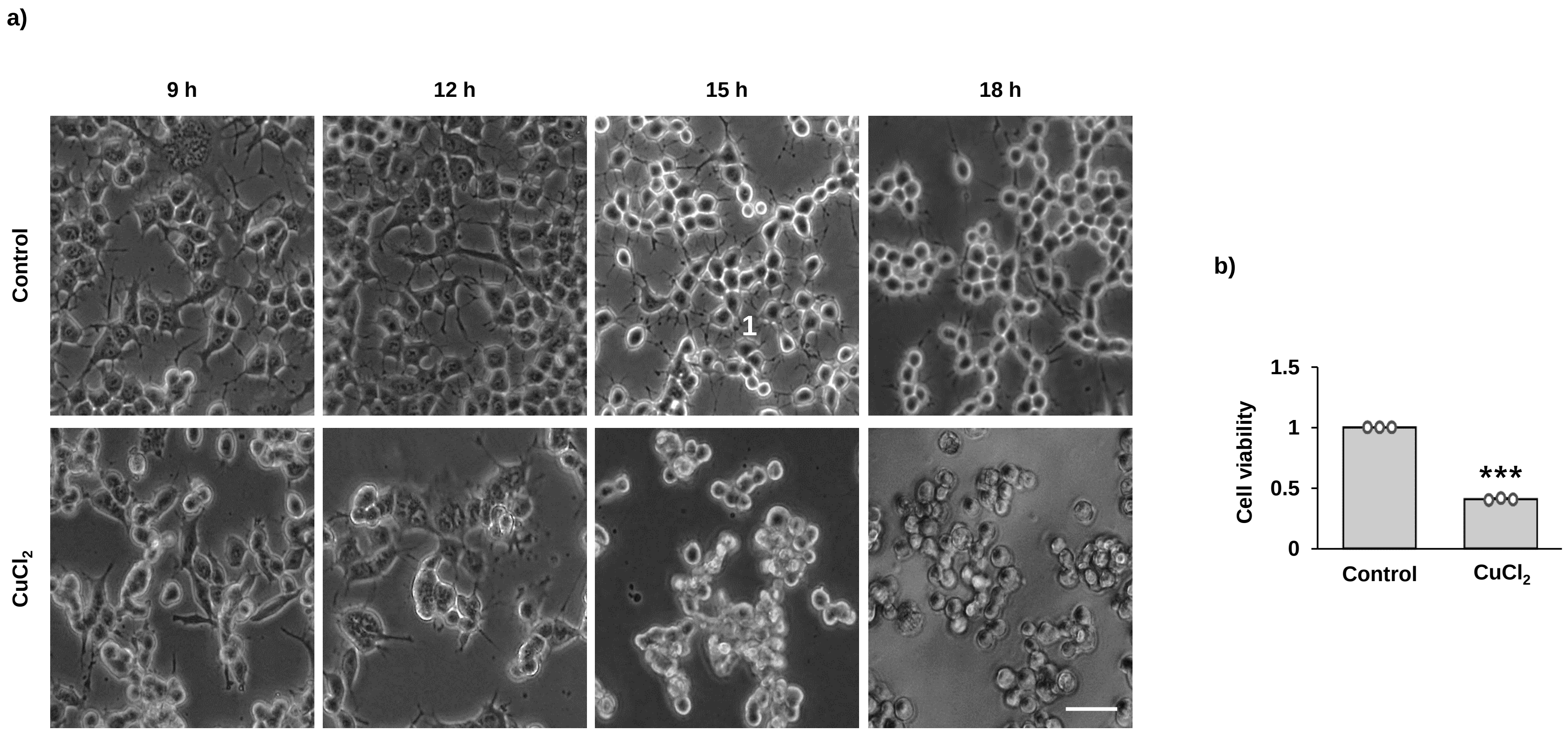

Supplementary figure 1. Phase-contrast photomicrographs and MTT reduction assay following $CuCl_2$ treatment. MN9D neuronal cells were treated with or without 250 μM $CuCl_2$ for the indicated time periods. **(a)** Cells were then examined by phase-contrast microscopy. Scale bar represents 50 μm. **(b)** MTT reduction assay was performed to assess cell viability, which was expressed as percentage over untreated control cells (100%). Data are shown as the mean ± S.D. of three independent experiments. $^{***}P < 0.001$.

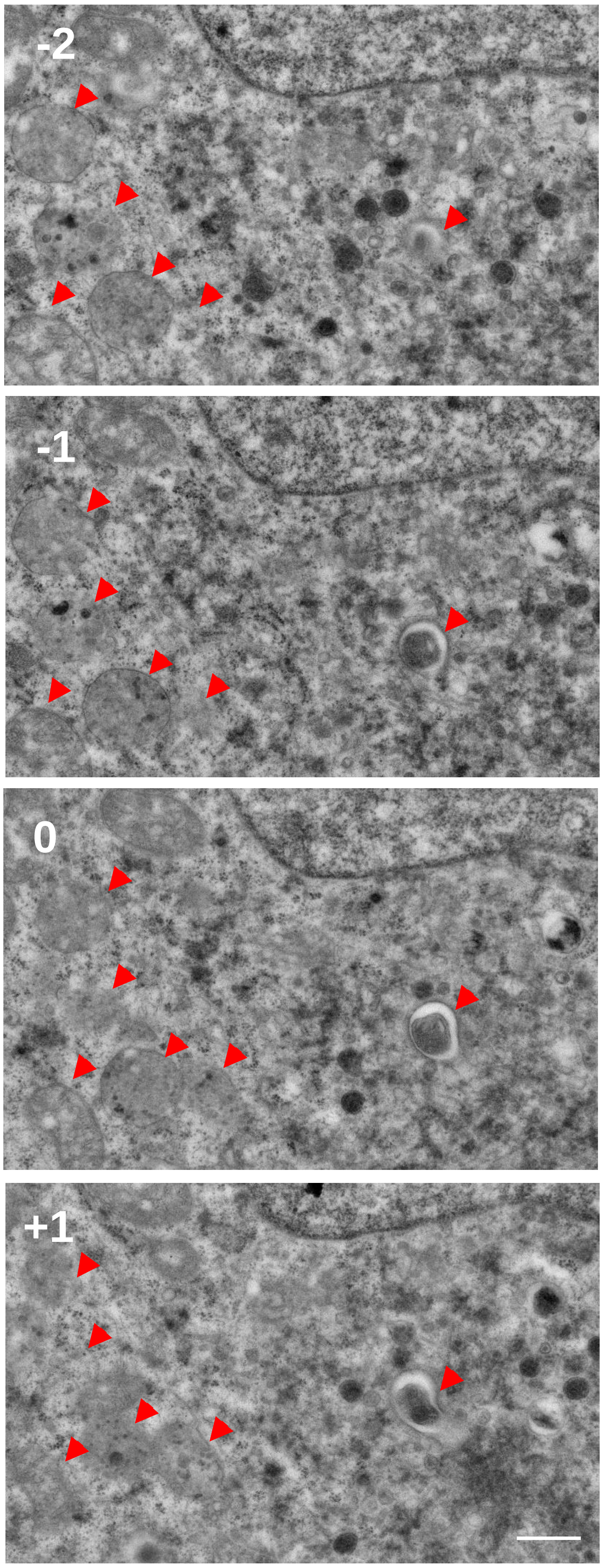

Supplementary figure 2. Serial section electron microscopy images of MN9D cells treated with $CuCl_2$. Electron micrographs were taken following treatment with 250 μM $CuCl_2$ for 15 h. Serial section images of the enlarged images show typical autophagic vacuoles (red arrows). Scale bar represents 500 nm.

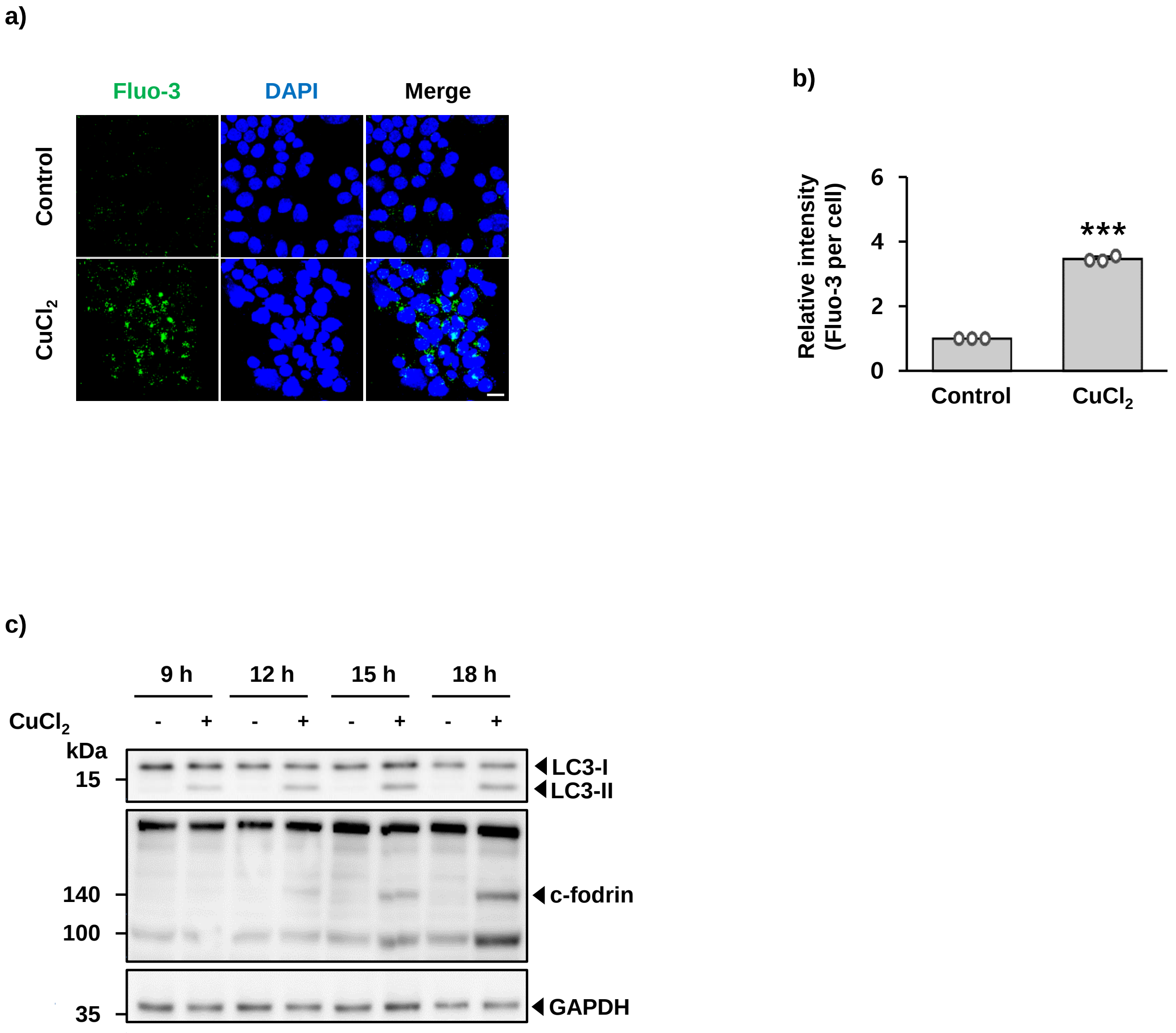

Supplementary figure 3. $CuCl_2$-induced surge of Ca^{2+}. (a-b) MN9D cells treated with or without 250 μM $CuCl_2$ for 15 h were stained with 3 μM Fluo-3 followed by counterstaining with Hoechst 33258 (DAPI). **(a)** Representative confocal images are provided. Scale bar represents 20 μm. **(b)** The relative intensity of Fluo-3 per cell was quantified using ImageJ software. Data are shown as the mean ± S.D. of three independent experiments. $^{***}P < 0.001$. **(c)** MN9D cells were treated with or without 250 μM $CuCl_2$ for the indicated time periods. Immunoblot analyses were performed using anti-LC3 or anti-Fodrin antibody. Calpain-cleaved form of fodrin (c-fodrin) is marked by arrowhead. Anti-GAPDH antibody was utilized as a loading control.

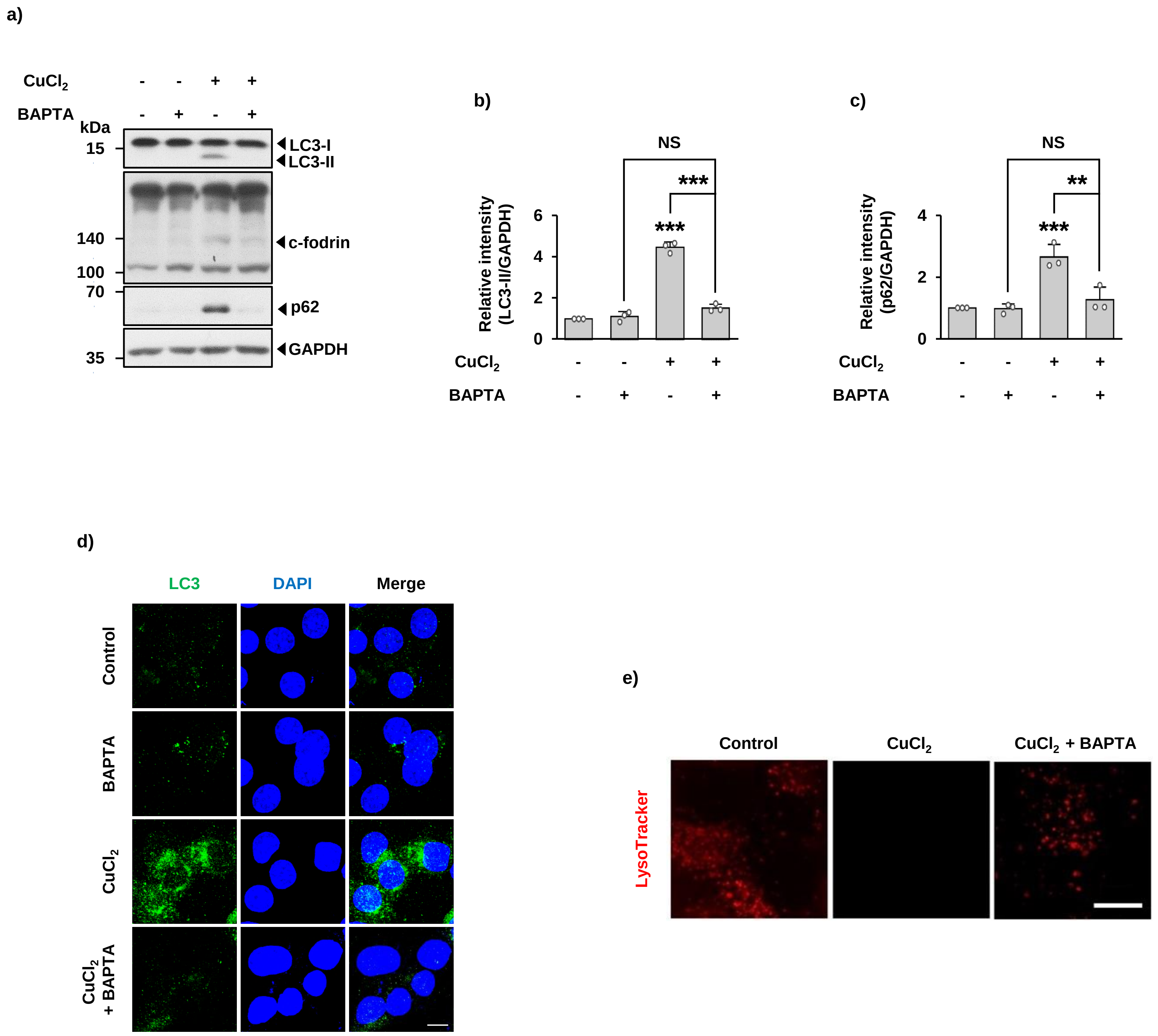

Supplementary figure 4. Ca^{2+} chelator-mediated restoration of $CuCl_2$-induced dysregulated autophagic flux. MN9D cells were incubated with 250 μM $CuCl_2$ for 15 h in the presence or absence of 30 min pre-treatment with 10 μM BAPTA-AM. **(a)** Immunoblot analyses were performed using anti-LC3, anti-fodrin, or anti-p62 antibody. Representative blots are provided. **(b-c)** The relative intensities of LC3-II (b) and p62 (c) were measured using ImageJ software, normalized by the intensity of GAPDH signal, and expressed as fold change relative to untreated control (value = 1). Data are shown as the mean ± S.D. of three independent experiments. Two-way ANOVA followed by Tukey's post hoc test was performed. $^{**}P < 0.01$; $^{***}P < 0.001$; NS, not significant. **(d)** Immunocytochemical localization analysis was performed using anti-LC3 (green) followed by counterstaining with Hoechst 33258 (blue). Representative confocal images are provided. Scale bar represents 10 μm. **(e)** Fluorescence images of LysoTracker Red were acquired using confocal microscopy. Scale bar represents 10 μm.

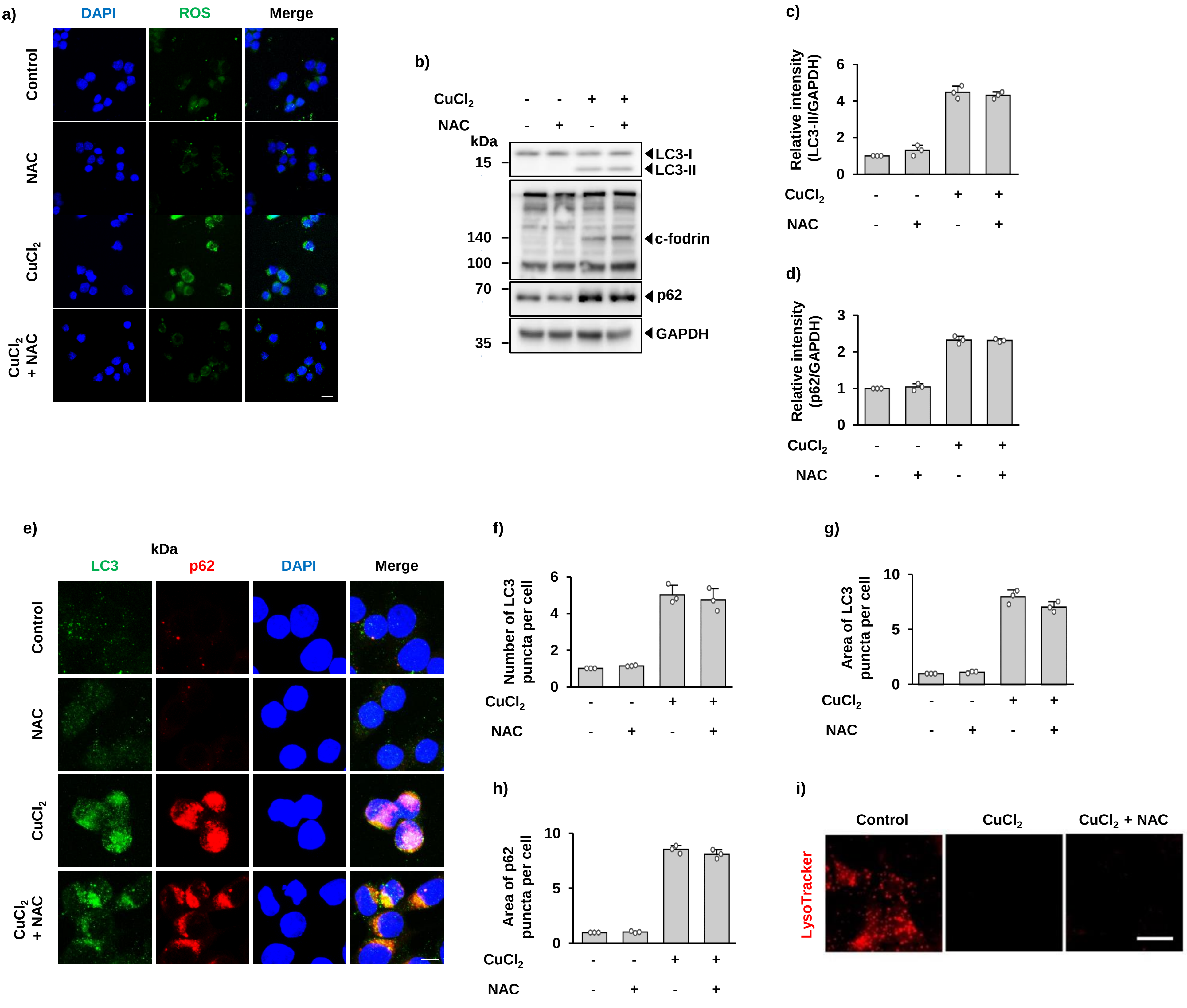

Supplementary figure 5. Absence of protective effect of antioxidant NAC against $CuCl_2$-induced dysregulated autophagic flux. MN9D cells were treated with or without 250 μM $CuCl_2$ for 15 h in the presence or absence of NAC (5 mM). **(a)** After drug treatment, cells were loaded with 3 μM CM-H2DCFDA for 30 min. Measurement of DCF fluorescence was performed by confocal microscopy. Representative images are provided. Scale bar represents 20 μm. **(b)** Immunoblot analyses were performed using the indicated antibodies. **(c-d)** The relative intensities of LC3-II (c) and p62 (d) signals were measured using ImageJ software, normalized by the intensity of GAPDH signal, and expressed as fold change relative to untreated control (value = 1). Data are shown as the mean ± S.D. of three independent experiments. Two-way ANOVA was performed ((c) Two-way ANOVA P value is 0.142497, and (d) Two-way ANOVA P value is 0.539701). **(e)** Immunocytochemical analyses were performed using anti-LC3 or anti-p62 antibody followed by counterstaining with Hoechst 33258. Scale bar represents 10 μm. **(f-h).** The number (f) and area (g) of LC3 and area of p62 (h) puncta per cell were quantified using ImageJ software. Data are shown as the mean ± S.D. of three independent experiments. Two-way ANOVA was performed ((f) Two-way ANOVA P value is 0.405501, (g) Two-way ANOVA P value is 0.050290 and (h) Two-way ANOVA P value is 0.176320). **(i)** Fluorescence images of LysoTracker Red were acquired by confocal microscopy. Representative images are provided. Scale bar represents 10 μm.
